# PIAS1-mediated SUMOylation of influenza A virus PB2 restricts viral replication and virulence

**DOI:** 10.1371/journal.ppat.1010446

**Published:** 2022-04-04

**Authors:** Guangwen Wang, Yuhui Zhao, Yuan Zhou, Li Jiang, Libin Liang, Fandi Kong, Ya Yan, Xuyuan Wang, Yihan Wang, Xia Wen, Xianying Zeng, Guobin Tian, Guohua Deng, Jianzhong Shi, Liling Liu, Hualan Chen, Chengjun Li

**Affiliations:** State Key Laboratory of Veterinary Biotechnology, Harbin Veterinary Research Institute, Chinese Academy of Agricultural Sciences, Harbin, The People’s Republic of China; Erasmus Medical Center, NETHERLANDS

## Abstract

Host defense systems employ posttranslational modifications to protect against invading pathogens. Here, we found that protein inhibitor of activated STAT 1 (PIAS1) interacts with the nucleoprotein (NP), polymerase basic protein 1 (PB1), and polymerase basic protein 2 (PB2) of influenza A virus (IAV). Lentiviral-mediated stable overexpression of PIAS1 dramatically suppressed the replication of IAV, whereas siRNA knockdown or CRISPR/Cas9 knockout of PIAS1 expression significantly increased virus growth. The expression of PIAS1 was significantly induced upon IAV infection in both cell culture and mice, and PIAS1 was involved in the overall increase in cellular SUMOylation induced by IAV infection. We found that PIAS1 inhibited the activity of the viral RNP complex, whereas the C351S or W372A mutant of PIAS1, which lacks the SUMO E3 ligase activity, lost the ability to suppress the activity of the viral RNP complex. Notably, the SUMO E3 ligase activity of PIAS1 catalyzed robust SUMOylation of PB2, but had no role in PB1 SUMOylation and a minimal role in NP SUMOylation. Moreover, PIAS1-mediated SUMOylation remarkably reduced the stability of IAV PB2. When tested in vivo, we found that the downregulation of Pias1 expression in mice enhanced the growth and virulence of IAV. Together, our findings define PIAS1 as a restriction factor for the replication and pathogenesis of IAV.

## Author summary

SUMOylation appears to be an important posttranslational modification mechanism of proteins, including viral proteins. In the present study, we found that the SUMO E3 ligase PIAS1 interacts with the PB2, PB1, and NP proteins of the RNP complex of IAV. PIAS1 expression was found to suppress the viral RNP complex activity. Mechanistically, the SUMO E3 ligase activity of PIAS1 led to robust SUMOylation of IAV PB2, but had no or a minimal effect on the SUMOylation of PB1 and NP, respectively, and PIAS1-mediated SUMOylation significantly decreased the stability of PB2. The expression of PIAS1 was markedly induced upon IAV infection in cell culture and mice, indicating that PIAS1 is actively involved and biologically important in the inhibition of IAV replication. Of note, the role of Pias1 in restricting the replication and virulence of IAV was directly verified in *Pias1*^*+/-*^ mice. Our findings thus identify a SUMO E3 ligase that interacts with and SUMOylates IAV PB2, thereby leading to reduced virus replication and virulence in vitro and in vivo.

## Introduction

Influenza A virus (IAV) is a widespread zoonotic pathogen that can infect various host species, including birds, lower mammals, and humans [1,2]. Although global efforts have been made to respond to, prevent, and control the threat posed by IAVs, seasonal influenza epidemics and occasional pandemics continue to challenge public health. In addition, increasing numbers of subtypes of avian influenza viruses, such as H5N1 and H7N9, have acquired the ability to cross the species barrier to infect and kill humans [3–5], raising concerns of the evolution of new pandemic viruses. It is therefore imperative that we understand the mechanisms of viral pathogenesis and host defense in order to develop better countermeasures.

The genome of IAV comprises eight single-stranded negative-sense RNA segments, encoding ten essential proteins as well as up to eight accessary proteins [6]. However, compared with its host, the genome and proteome of IAV are too simple to complete virus replication unaided. Therefore, IAV must rely on its host’s cellular machinery and factors to support its life cycle and become pathogenic [7–11]. In turn, the host encodes restriction factors (e.g., IFITM3, LSD1, MOV10, PKP2, PLSCR1, TRIM25, TRIM32, TRIM35, and TUFM) [12–20] to suppress the replication and virulence of IAVs. The transcription and replication of the IAV genome take place in the nucleus of virus-infected cells [21,22], catalyzed by the viral ribonucleoprotein (RNP) complex, which is composed of polymerase basic protein 2 (PB2), polymerase basic protein 1 (PB1), polymerase acidic protein (PA), and nucleoprotein (NP) [23]. To fulfill the function of transcribing and replicating the viral genome, the newly synthesized viral polymerases and NP must assemble into a complete complex with the viral RNAs. The central role of the vRNP complex in the virus life cycle makes it a key target of the host defense system.

SUMOylation, the process of conjugating a small ubiquitin-like modifier (SUMO) to target proteins, has been recognized as an important posttranslational modification mechanism. SUMOylation is primarily involved in processes that occur in the nucleus [24,25], and leads to the modification of the localization, activity, stability, and function of target proteins [26]. In mammals, there are four members of the SUMO protein family: SUMO1, SUMO2, SUMO3, and SUMO4 [26,27]. SUMO1 is a 101-amino-acid protein of approximately 11.6 kDa. SUMO2 and SUMO3 differ from each other by only three N-terminal residues, and together they share only approximately 47% homology with SUMO1. SUMO1, SUMO2, and SUMO3 can covalently attach to the acceptor lysines on target substrates. SUMO4 is very similar to SUMO2/3. However, SUMO4 seems to be blocked in its maturation and is probably non-conjugated to substrates under physiological conditions. The SUMOylation cascade involves the SUMO E1 activating enzyme SAE1/SAE2, SUMO E2 conjugase Ubc9, and a limited number of SUMO E3 ligases.

Protein inhibitor of activated STAT 1 (PIAS1) was initially identified as an inhibitor of the STAT1 signaling pathway [28], and functions as a negative regulator of innate immunity [29,30]. However, many studies have now revealed that PIAS1 is a multi-functional protein that is essential in a variety of biological processes, such as DNA repair [31], apoptosis [32–34], tissue development [35,36], differentiation [37–41], and tumorigenesis [42–44]. PIAS1 is involved in the posttranslational SUMO modification of numerous target proteins, including many vital transcription factors, such as p53 and Myc [43,45,46]. PIAS1 generally relies on its SUMO E3 ligase activity during these different biological processes [47]; however its interaction with various binding partners could also lead to a change in the localization of a target protein or affect the interaction of the target proteins with other proteins in a SUMOylation-independent manner [48,49]. PIAS1 has also been reported to be involved in the infection course of different viruses, such as Ebola virus (EBOV), Epstein-Barr virus (EBV), Herpes simplex virus 1 (HSV-1), and Vesicular stomatitis virus (VSV) [29,50–53].

In the present study, we found that PIAS1 interacts with three viral RNP components of IAV: NP, PB1, and PB2. SiRNA knockdown or CRISPR/Cas9 knockout of PIAS1 expression dramatically increased the replication titer of a range of IAVs, whereas overexpression of PIAS1 inhibited IAV propagation. PIAS1 expression was markedly induced in IAV-infected cells or mice, and the presence of PIAS1 was important for the overall increase in cellular SUMOylation induced by IAV infection. We further demonstrated that PIAS1 expression suppressed the viral RNP activity, which was dependent on the SUMO E3 ligase activity of PIAS1. The SUMO E3 ligase activity of PIAS1 had no effect on the stability of NP and PB1, but led to apparent degradation of PB2. Moreover, downregulation of Pias1 expression in *Pias1*^*+/-*^ mice resulted in enhanced virus replication and virulence. Our findings thus established that PIAS1 is a restriction factor for IAV infection and pathogenesis.

## Results

### PIAS1 interacts with multiple proteins in the RNP complex of IAV

We utilized the same yeast-two-hybrid (Y2H) approach, which has been previously described [19], to identify potential interacting partners of IAV NP. A high frequency clone encoding PIAS1 was obtained as a potential NP-interactor in the screen. The interaction of PIAS1 with NP was confirmed by co-transforming the bait plasmid pGBDT7-NP of A/Anhui/2/2005 (AH05, H5N1) virus and the prey plasmid pGADT7-PIAS1 into the Y2HGold yeast strain and growing the transformant on plates with special medium (S1 Fig).

**Fig 1:**
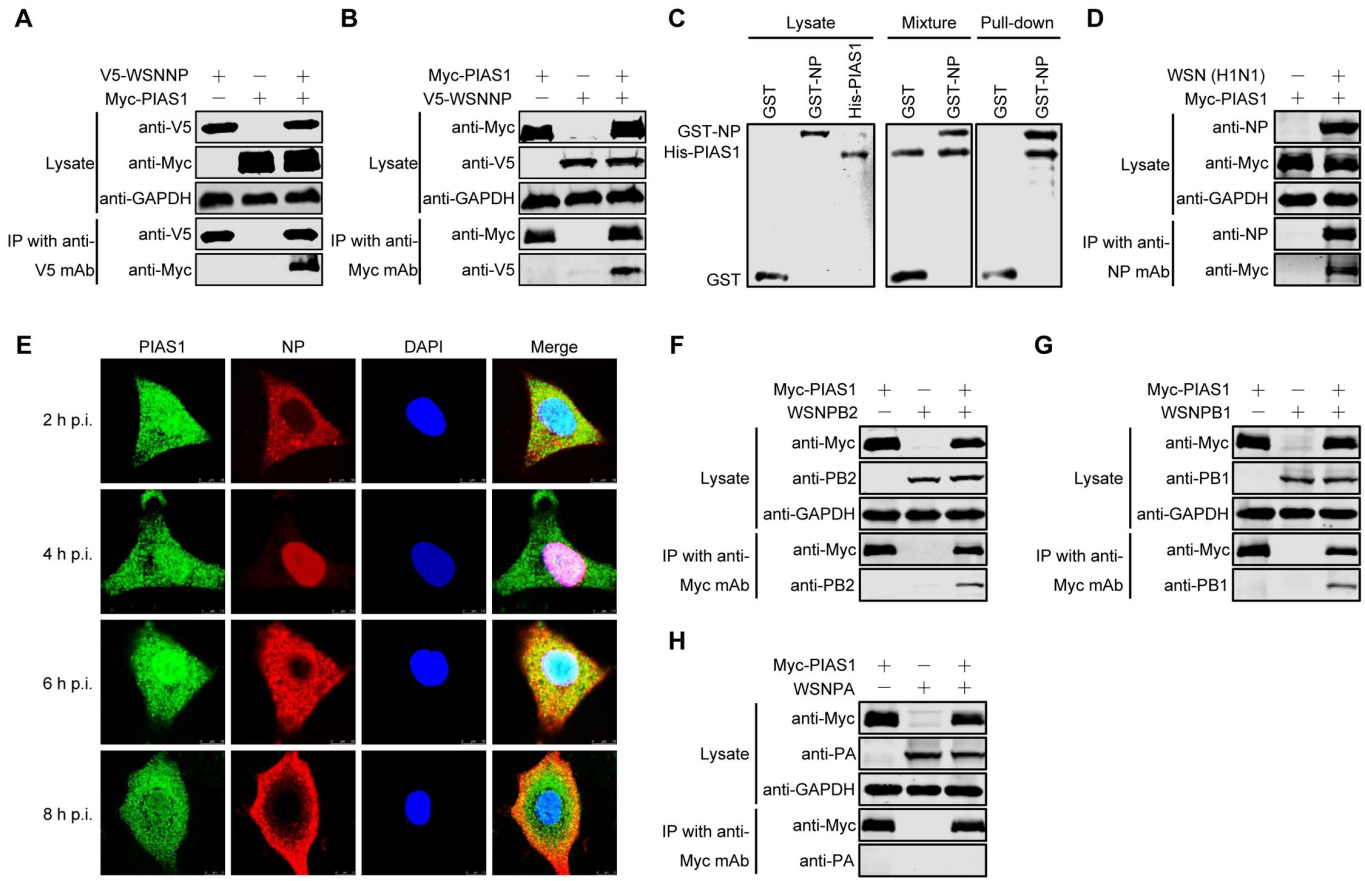
PIAS1 interacts with multiple proteins in the RNP complex of IAV. (A, B) Interaction of V5-WSNNP and Myc-PIAS1 in HEK293T cells by using a co-IP assay. HEK293T cells were individually transfected or co-transfected with plasmids expressing V5-WSNNP and Myc-PIAS1. Cell lysates were immunoprecipitated with a mouse anti-V5 mAb (A) or a mouse anti-Myc mAb (B), and subjected to western blotting with a rabbit anti-V5 pAb and a rabbit anti-Myc pAb for the detection of WSNNP and PIAS1, respectively. (C) Interaction of GST-WSNNP and His-PIAS1 by using a GST pull-down assay. His-tagged PIAS1 was expressed in *E*. *coli* BL21 (DE3) and purified by using Ni Sepharose Excel resin, and the GST or GST-NP protein was expressed in HEK293T cells and purified by using Glutathione Sepharose 4 Fast Flow. An equal amount of purified PIAS1 was mixed with the Glutathione Sepharose 4 Fast Flow samples that bind GST or GST-NP. After rocking and washing, the mixed samples were separated by SDS-PAGE and stained with Coomassie blue. (D) Interaction of IAV NP and PIAS1 in virus-infected cells. HEK293T cells were transfected for 24 h to express Myc-PIAS1, and were then infected with WSN (H1N1) virus (MOI = 5). At 30 h p.i., cell lysates were immunoprecipitated with a mouse anti-NP mAb, followed by western blotting with a rabbit anti-NP pAb and a rabbit anti-Myc pAb. (E) Co-localization of IAV NP and PIAS1 in A549 cells infected with WSN (H1N1) virus. A549 cells were infected with WSN (H1N1) virus (MOI = 5). At 2, 4, 6, and 8 h p.i., the infected cells were fixed and stained with a mouse anti-NP mAb and a rabbit anti-PIAS1 pAb, followed by incubation with Alexa Fluor 633 goat anti-mouse IgG (H+L) (red) and Alexa Fluor 488 donkey anti-rabbit IgG (H+L) (green). The nuclei were stained with DAPI. (F-H) Co-IP assay to examine the interactions between Myc-PIAS1 and PB2, PB1, and PA of WSN (H1N1) virus in HEK293T cells. HEK293T cells were individually transfected or co-transfected with plasmids expressing WSNPB2, WSNPB1, WSNPA, and Myc-PIAS1. Cell lysates were immunoprecipitated with a mouse anti-Myc mAb and were subjected to western blotting with a rabbit anti-PB2 pAb (F), a rabbit anti-PB1 pAb (G), a rabbit anti-PA pAb (H), and a rabbit anti-Myc pAb (F-H) for the detection of PB2, PB1, PA, and PIAS1, respectively.

To determine whether NP interacts with PIAS1 in mammalian cells, HEK293T cells were transfected with V5-tagged NP of A/WSN/33 (WSN, H1N1) virus and Myc-tagged PIAS1 constructs, either alone or together. Cell lysates were subject to immunoprecipitation with a mouse anti-V5 monoclonal antibody (mAb), followed by western blotting with a rabbit anti-V5 or anti-Myc polyclonal antibody (pAb). Myc-tagged PIAS1 was co-immunoprecipitated with V5-tagged WSNNP only when they were co-expressed (Fig 1A). Conversely, V5-tagged WSNNP was also co-immunoprecipitated with Myc-tagged PIAS1 when the co-IP experiment was performed with a mouse anti-Myc mAb (Fig 1B). Together, these results indicate that PIAS1 interacts with IAV NP in transiently transfected mammalian cells.

Next, we performed a GST pull-down experiment to determine whether NP and PIAS1 directly interact with each other. HEK293T cells were transfected individually with constructs expressing GST or GST-WSNNP. At 48 h post-transfection, the expressed GST or GST-WSNNP in the cell lysates were purified with Glutathione Sepharose 4 Fast Flow. His-tagged PIAS1 was expressed in *E*. *coli* BL21 (DE3) and purified by using Ni Sepharose Excel resin. An equal amount of purified PIAS1 was mixed with the Glutathione Sepharose 4 Fast Flow samples that bind GST or GST-NP. After rocking and washing, the mixed samples were separated by SDS-PAGE and stained with Coomassie blue. As shown in Fig 1C, purified PIAS1 was only pulled down by GST-WSNNP, and not GST alone, indicating that IAV NP directly binds to PIAS1 in vitro.

To further explore the interaction between NP and PIAS1 during IAV infection, HEK293T cells were transfected for 24 h to express Myc-PIAS1, and then subjected to infection with WSN (H1N1) virus at an MOI of 5. At 30 h post-infection (p.i.), the cell lysates were immunoprecipitated with a mouse anti-NP mAb, and the presence of NP and PIAS1 in the immunoprecipitates was revealed by western blotting. We found that IAV NP efficiently interacted with PIAS1 during virus replication (Fig 1D).

To reveal the localization of NP and PIAS1 during virus infection, we performed immunostaining and confocal microscopy analysis. A549 cells infected with WSN (H1N1) virus at an MOI of 5 were fixed at 2, 4, 6, or 8 h p.i., followed by immunostaining with anti-NP and anti-PIAS1 antibodies. Confocal microscopy showed that PIAS1 was distributed in both the cytoplasm and nucleus (Fig 1E). Most of the NP was localized in the cytoplasm at 2 h p.i., accumulated in the nucleus at 4 h p.i., was exported to the cytoplasm at 6 h p.i., and gathered around the plasma membrane at 8 h p.i. Of note, PIAS1 partially colocalized with NP during the shuttle of NP between the cytoplasm and nucleus at different stages of virus infection.

The above results indicate that PIAS1 interacts with IAV NP in both transfected and infected cells. Given that the RNP complex is a compact functional unit for the replication of IAV [54,55], we next attempted to investigate whether PIAS1 also interacts with other protein components of the RNP complex. To that end, we performed co-IP experiments in transiently transfected HEK293T cells and found that PB2 and PB1, but not PA, were also co-immunoprecipitated with Myc-tagged PIAS1 (Fig 1F–1H). Consistent with these data, we found that PIAS1 was also co-immunoprecipitated with PB2 and PB1, but not PA, during IAV infection (S2 Fig). We further investigated whether PIAS1 interacted with other internal (M1) or surface proteins (HA, NA and M2) of IAV by co-IP experiments, and found that PIAS1 did not interact with these structural components of IAV (S3 Fig). These data indicate that three components of the IAV RNP complex, that is, PB2, PB1, and NP, efficiently interact with cellular PIAS1.

### PIAS1 suppresses the replication of IAV in vitro

**Fig 2:**
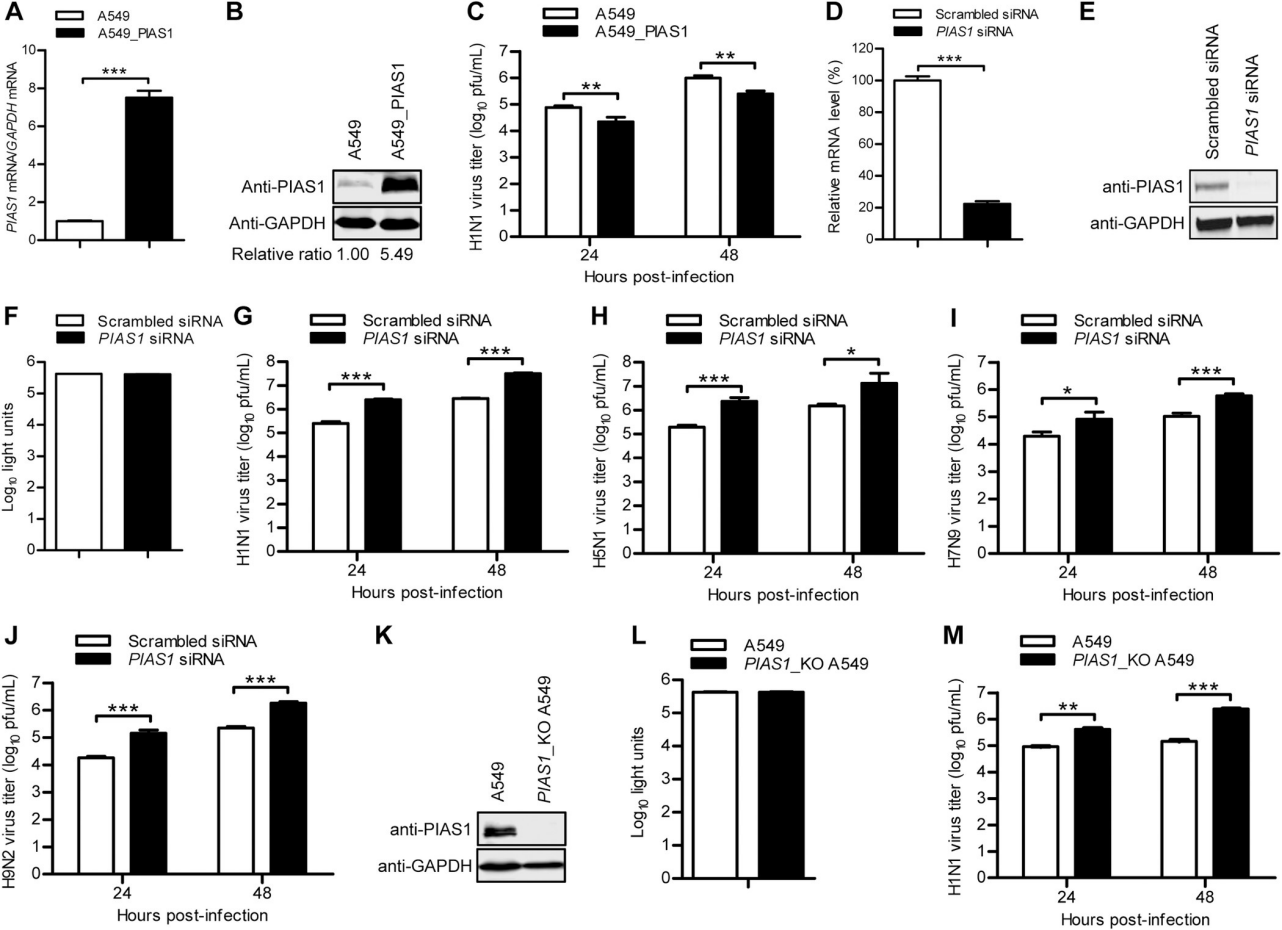
PIAS1 restricts IAV replication in vitro. (A, B) Establishment of a lentiviral-mediated PIAS1-overexpressing A549 cell line. The stable expression of PIAS1 was confirmed by quantitative reverse transcription PCR (RT-qPCR) (A) and western blotting with a rabbit anti-PIAS1 pAb (B). ***, *P* < 0.001. (C) The PIAS1-overexpressing or control A549 cells were infected with WSN (H1N1) virus (MOI = 0.01). Supernatants were collected at the indicated timepoints, and virus titers were determined by means of plaque assays on MDCK cells. **, *P* < 0.01. (D, E) siRNA knockdown of PIAS1 in A549 cells. A549 cells were transfected with siRNA targeting *PIAS1* or with scrambled siRNA for 48 h. Cell lysates were subjected to RT-qPCR (D) or western blotting with a rabbit anti-PIAS1 pAb (E) to confirm the downregulation of PIAS1. ***, *P* < 0.001. (F) Cell viability of siRNA-treated A549 cells as in (D, E) was determined by using a CellTiter-Glo assay. (G to J) Virus replication in siRNA-treated A549 cells as in (D, E). *PIAS1* siRNA- or scrambled siRNA-transfected A549 cells were infected with WSN (H1N1) (MOI = 0.01) (G), AH05 (H5N1) (MOI = 0.1) (H), AH13 (H7N9) (MOI = 0.1) (I), or SH13 (H9N2) (MOI = 0.1) (J) virus. Supernatants were collected at the indicated timepoints, and virus titers were determined by means of plaque assays on MDCK cells. *, *P* < 0.05, ***, *P* < 0.001. (K) Knockout of PIAS1 in *PIAS1*_KO A549 cells was confirmed by western blotting with a rabbit anti-PIAS1 pAb. (L) Cell viability of *PIAS1*_KO A549 cells was determined by using the CellTiter-Glo assay. (M) Virus replication in *PIAS1*_KO A549 cells. *PIAS1*_KO or control A549 cells were infected with WSN (H1N1) (MOI = 0.01) virus. Supernatants were collected at the indicated timepoints, and virus titers were determined by means of plaque assays on MDCK cells. **, *P* < 0.01, ***, *P* < 0.001.

We demonstrated that PIAS1 interacts with PB2, PB1, and NP of the RNP complex of IAV. To determine the role of PIAS1 in the regulation of IAV replication, we first established a lentiviral vector-based A549 cell line stably overexpressing PIAS1 to examine the effect of PIAS1 upregulation on IAV replication. Quantitative reverse transcription PCR (RT-qPCR) and western blotting showed that PIAS1 overexpression produced a 7.5-fold increase in mRNA transcript abundance and a 5.49-fold increase in protein expression compared with the empty lentivirus-transduced control cell line (Fig 2A and 2B). The PIAS1-overexpressing or control A549 cells were infected with WSN (H1N1) virus at an MOI of 0.01, and the culture supernatant was collected at 24 and 48 h p.i. for titration of infectious virus by use of plaque assays. As shown in Fig 2C, stable overexpression of PIAS1 led to a 3.5- and 4-fold decrease in viral titers at 24 and 48 h p.i., respectively.

Next, we examined the effect of PIAS1 downregulation on virus replication by using siRNA knockdown. A549 cells treated with specific siRNA targeting *PIAS1* or scrambled siRNA were infected with WSN (H1N1) virus at an MOI of 0.01, and the culture supernatant was collected at 24 and 48 h p.i. for virus titration. *PIAS1*-specific siRNA treatment efficiently reduced the expression of PIAS1 compared with scrambled siRNA treatment (Fig 2D and 2E), and PIAS1 knockdown had no obvious effect on the viability of the siRNA-treated cells (Fig 2F). As shown in Fig 2G, *PIAS1* knockdown led to a 10.3- and 11.3-fold increase in WSN (H1N1) virus titer at 24 and 48 h p.i., respectively. Similarly, siRNA knockdown of PIAS1 expression led to 11.9-/8.7-, 4.2-/5.7-, and 7.8-/8.1-fold increases in the growth titers of AH05 (H5N1), A/Anhui/1/2013 (AH13, H7N9), and A/chicken/Shanghai/SC197/2013 (SH13, H9N2) virus at 24/48 h p.i. (Fig 2H–2J), demonstrating that PIAS1 downregulation enhances the replication of a broad range of IAVs.

We then generated a *PIAS1* knockout (*PIAS1*_KO) A549 cell line by using the CRISPR/Cas9 system (Fig 2K). Knockout of PIAS1 expression had no major effect on cell viability (Fig 2L). The titers of WSN (H1N1) virus grown on *PIAS1*_KO A549 cells increased 4.5 and 16.8 fold compared with those of virus grown on control cells at 24 and 48 h p.i., respectively (Fig 2M). To investigate the effect of PIAS1 knockout on viral protein expression, we infected *PIAS1*_KO A549 cells with WSN (H1N1) virus at an MOI of 5. Confocal microscopy showed during the course of WSN (H1N1) infection, between 2 and 8 h p.i., the expression of viral NP, an indicator of virus replication, was dramatically enhanced in *PIAS1*_KO A549 cells compared with that in control cells (S4 Fig). These data further confirm that PIAS1 negatively modulates IAV replication.

Collectively, these results indicate that PIAS1 functions as a host restriction factor against IAV replication.

### IAV infection induces the expression of PIAS1 in vitro and in vivo

To investigate whether the dynamics of PIAS1 expression is affected during IAV infection, we infected A549 cells with WSN (H1N1) virus at an MOI of 0.1 and determined the expression of PIAS1 by western blotting at 0, 12, and 24 h p.i. We found that the level of PIAS1 expression gradually increased as the infection progressed (S5A Fig). To investigate whether the increase in PIAS1 expression upon IAV infection is regulated by type I interferon (IFN), A549 cells were treated with or without 25 pg/mL IFN-β for 24 h. We found that the level of PIAS1 expression was unchanged by IFN-β treatment compared with that of control cells (S5B Fig). By contrast, the expression of MX1 and IFITM3, two well-known interferon-stimulated genes, was significantly triggered by IFN-β treatment. These data indicate that the expression of PIAS1 in vitro is induced solely by IAV infection, and is not dependent on IFN-β treatment. To determine whether the increase in PIAS1 expression also occurred in vivo, 6-week-old C57BL/6J mice were infected with 10^5^ PFU of WSN (H1N1) and AH13 (H7N9), 10^2^ PFU of AH05 (H5N1), 10^6^ PFU of SH13 (H9N2) virus, or were mock-infected with PBS. The lungs of the infected mice were collected on day 3 p.i., and lung homogenates were western blotted with an anti-PIAS1 pAb. We found that for the four IAV strains, the level of Pias1 increased by 6.0, 12.6, 6.1, and 15.1 fold, respectively, in the lungs of IAV-infected mice compared with mock-infected mice (S5C Fig). These results demonstrate that the expression of PIAS1 is actively induced upon IAV infection in vitro and in vivo.

### PIAS1 is involved in gross SUMOylation during IAV infection

**Fig 3:**
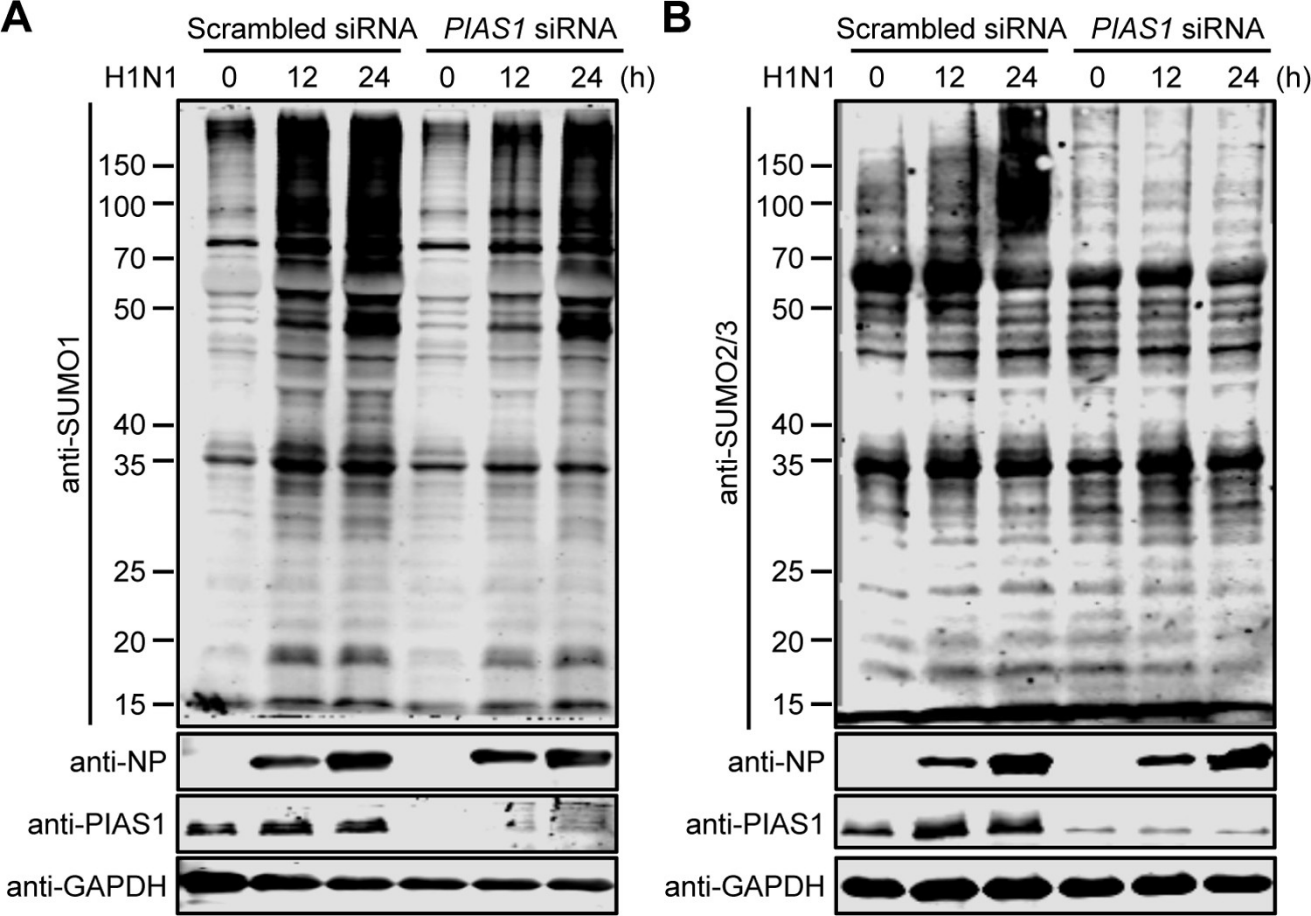
PIAS1 regulates the gross SUMOylation induced by IAV infection. A549 cells treated with specific siRNA targeting *PIAS1* or scrambled siRNA were infected with WSN (H1N1) virus at an MOI of 0.01. The overall cellular SUMOylation level was assessed at 0, 12, and 24 h p.i. by western blotting with an anti-SUMO1 (A) or anti-SUMO2/3 (B) pAb.

IAV infection triggers an increase in the abundance of proteins modified by both SUMO1 and SUMO2/3 [56]. To explore whether PIAS1 is a contributor to the overall increase in SUMOylation during IAV infection, A549 cells treated with specific siRNA targeting *PIAS1* or scrambled siRNA were infected with WSN (H1N1) virus at an MOI of 0.01, and then the overall SUMOylation level was assessed at 0, 12, and 24 h p.i. by using an anti-SUMO1 or SUMO2/3 pAb. We found that IAV infection triggered an increase in the abundance of proteins modified by both SUMO1 and SUMO2/3, and that knockdown of PIAS1 expression reduced the overall level of cellular SUMOylation during IAV infection (Fig 3A and 3B). These data indicate that PIAS1 is involved in the gross cellular SUMOylation induced by IAV infection.

### PIAS1 inhibits viral RNA transcription and replication

**Fig 4:**
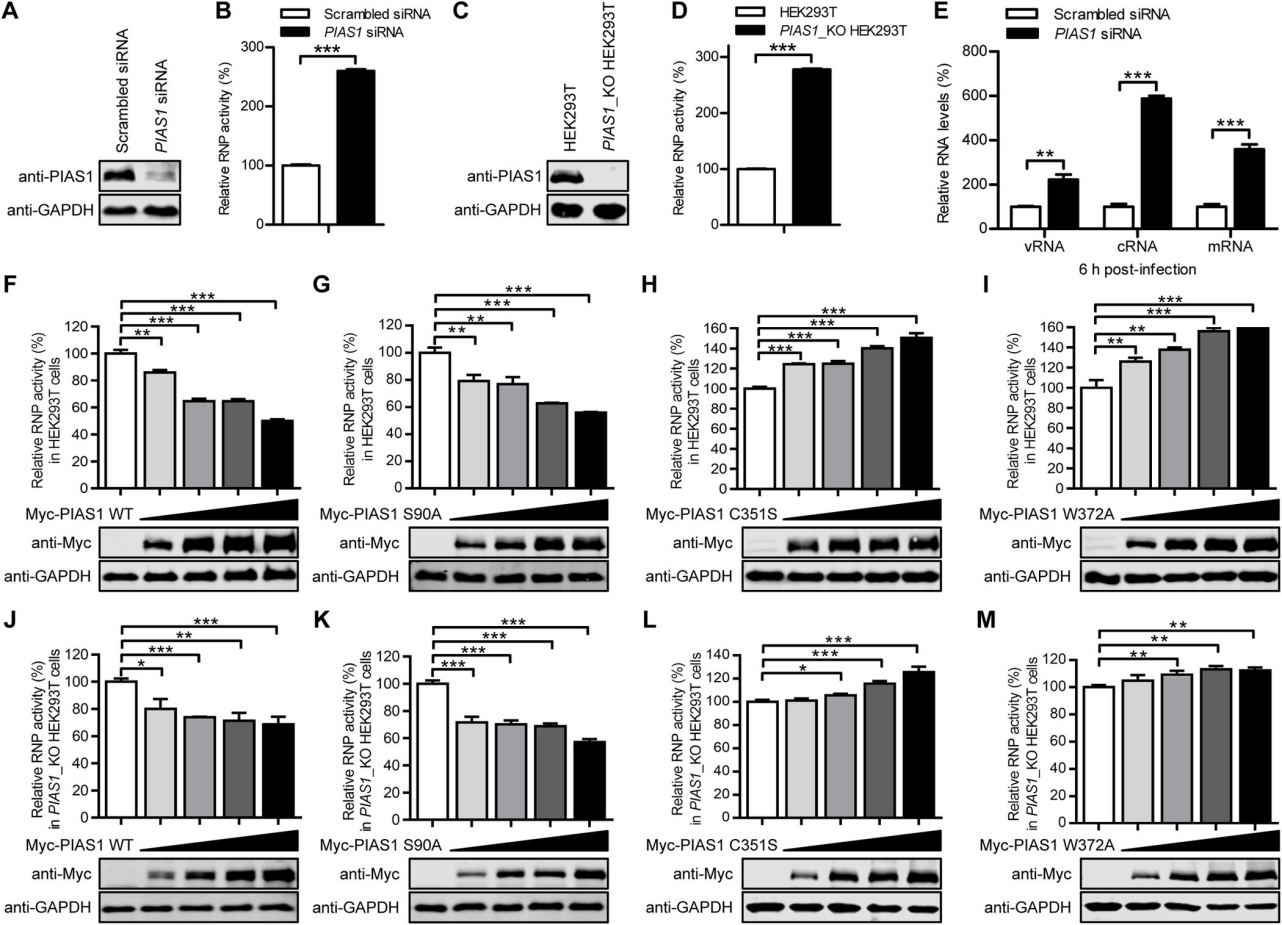
PIAS1 relies on its SUMO E3 ligase activity to suppress IAV transcription and replication. (A) siRNA knockdown of PIAS1 in HEK293T cells. HEK293T cells were transfected with *PIAS1* siRNA or scrambled siRNA for 48 h. Knockdown of PIAS1 expression was confirmed by western blotting with a rabbit anti-PIAS1 pAb. (B) Minigenome assay in siRNA-treated HEK293T cells to determine the effect of endogenous PIAS1 on viral RNP activity. HEK293T cells treated with siRNA as in (A) were transfected with plasmids for the expression of four viral RNP proteins (WSNPB2, WSNPB1, WSNPA, and WSNNP), together with pHH21-SC09NS F-Luc and pRL-TK. Thirty-six hours later, a dual-luciferase assay was performed in which the relative firefly luciferase activity was normalized to the internal control, Renilla luciferase activity. ***, *P* < 0.001. (C) Establishment of a *PIAS1*_KO HEK293T cell line. The knockout of PIAS1 was confirmed by western blotting with a rabbit anti-PIAS1 pAb. (D) Minigenome assay in *PIAS1*_KO HEK293T cells to determine the effect of endogenous PIAS1 on viral RNP activity. The minigenome assay was performed in *PIAS1*_KO HEK293T cells as in (B). ***, *P* < 0.001. (E) RT-qPCR analysis to determine the effect of PIAS1 knockdown on the transcription and replication of viral RNAs. A549 cells were transfected with siRNA targeting *PIAS1* or with scrambled siRNA for 48 h, followed by infection with WSN (H1N1) virus (MOI = 5). Total RNA was extracted at 6 h p.i. by using TRIzol reagent, and the levels of *NP*-specific vRNA, cRNA, and mRNA were analyzed by RT-qPCR and then normalized to *GAPDH* mRNA. The values shown are standardized to the corresponding RNA expression level in the scrambled siRNA-treated A549 cells (100%). **, *P* < 0.01, ***, *P* < 0.001. (F-I) Minigenome assay in HEK293T cells to examine the effect of exogenously expressed wild-type PIAS1 and PIAS1 mutants on viral RNP activity. HEK293T cells were transfected with plasmids for the expression of four viral RNP proteins and increasing amounts of Myc-PIAS1 or Myc-PIAS1 mutant, together with pHH21-SC09NS F-Luc and pRL-TK. At 36 h post-transfection, a dual-luciferase assay was performed in which the relative firefly luciferase activity was normalized to the Renilla luciferase activity. **, *P* < 0.01, ***, *P* < 0.001. (J-M) Minigenome assay in *PIAS1*_KO HEK293T cells to examine the effect of exogenously expressed wild-type PIAS1 and PIAS1 mutants on viral RNP activity. The minigenome assay was performed in *PIAS1*_KO HEK293T cells as in (F-I). *, *P* < 0.05, **, *P* < 0.01, ***, *P* < 0.001.

Having found that PIAS1 interacts with three components of the viral RNP complex and restricts IAV replication, we hypothesize that PIAS1 protein expression may affect the transcription and replication of the viral genome. To test this hypothesis, we first performed a minigenome assay in HEK293T cells that were treated with *PIAS1*-specific siRNA or scrambled siRNA for 12 h, followed by transfection with constructs expressing the viral RNP proteins (PB2, PB1, PA, NP) of WSN (H1N1) virus, a firefly luciferase reporter flanked with the packaging signals of the NS segment, and an internal Renilla luciferase control. The viral RNP activity was evaluated by measuring the luciferase activity at 36 h post-transfection. The results revealed a 2.6-fold increase in the viral RNP activity in *PIAS1*-specific siRNA- versus scrambled siRNA-treated cells (Fig 4A and 4B). To confirm these results, we generated a *PIAS1*_KO HEK293T cell line by using the CRISPR/Cas9 system (Fig 4C). HEK293T cells and *PIAS1*_KO HEK293T cells were transfected with the constructs for the minigenome assay, and the viral RNP activity was evaluated by measuring the luciferase activity at 36 h post-transfection. We found that the viral RNP activity increased 2.7-fold in *PIAS1*_KO versus wild-type HEK293T cells (Fig 4D). Together, these data indicate that the expression of endogenous PIAS1 inhibits the RNP activity of IAV.

To further explore which step, transcription or replication, is inhibited by the expression of PIAS1, A549 cells treated with *PIAS1*-specific siRNA or scrambled siRNA were infected with WSN (H1N1) virus at an MOI of 5, followed by RT-qPCR analysis to measure the levels of the three species of viral RNA at 6 h p.i. The levels of vRNA, cRNA, and mRNA increased by 2.2-, 5.9- and 3.6-fold, respectively, in PIAS1-knockdown cells compared with scrambled siRNA-treated cells (Fig 4E). This result indicates that both transcription and replication of the viral genome are inhibited by the expression of PIAS1 protein.

### The SUMO E3 ligase activity of PIAS1 is essential for its inhibitory effect on viral RNP activity

To identify the biological property of PIAS1 associated with its inhibitory effect on the RNP activity of IAV, we first assessed the effect of PIAS1 overexpression on viral RNP activity. HEK293T cells were transfected with the constructs for the minigenome assay, together with gradually increasing amounts of the Myc-PIAS1 expression construct. At 36 h post-transfection, the luciferase activity of the cell lysates was measured. We found that the overexpression of PIAS1 reduced the viral RNP activity in a dose-dependent manner (Fig 4F).

Ser90 phosphorylation of PIAS1 is essential for PIAS1-mediated repression of inflammatory gene activation [30]. To assess whether the inhibitory effect of PIAS1 on the RNP complex activity of IAV is dependent on Ser90 phosphorylation, HEK293T cells were transfected with the minigenome constructs, along with gradually increasing amounts of the Myc-PIAS1 S90A construct. The S90A PIAS1 mutant inhibited the viral RNP activity to a similar degree as wild-type PIAS1 (Fig 4G), indicating that Ser90 phosphorylation is not essential for PIAS1 to inhibit the RNP complex activity of IAV.

PIAS1 functions as a SUMO E3 ligase, whose SP-RING domain interacts with the SUMO E2 conjugase Ubc9 and is essential for SUMOylation reactions [36]. The SUMOylation mutations of C351S or W372A in PIAS1 abolish its SUMOylation activity [30,57]. To determine whether the SUMO E3 ligase activity is essential for PIAS1 to inhibit the RNP complex activity of IAV, we transfected HEK293T cells with the minigenome constructs, along with gradually increasing amounts of the Myc-PIAS1 C351S or W372A construct. We found that compared with wild-type PIAS1, both the C351S and W372A mutants lost the ability to inhibit the RNP complex activity of IAV (Fig 4H and 4I). Instead, the expression of the C351S or W372A mutant led to increases in the RNP activity of IAV. These findings indicate that PIAS1 inhibits the IAV RNP complex activity in a SUMOylation-dependent manner.

To validate the role of the SUMO E3 ligase activity of PIAS1 in inhibiting the RNP activity of IAV, we performed the minigenome assay in *PIAS1*_KO HEK293T cells. We found that the complement of wild-type or the S90A mutant of PIAS1 in the *PIAS1*_KO cells significantly reduced the viral RNP complex activity compared with the vector control (Fig 4J and 4K). In contrast, the addition of the C351S or W372A mutant of PIAS1 resulted in no inhibitory effect on the viral RNP complex activity (Fig 4L and 4M). These results further confirm that the SUMO E3 ligase activity is essential for PIAS1 to suppress the RNP activity of IAV.

### PIAS1 mediates robust SUMOylation of PB2, but has no effect on SUMOylation of PB1 and has a minimal effect on SUMOylation of NP

**Fig 5:**
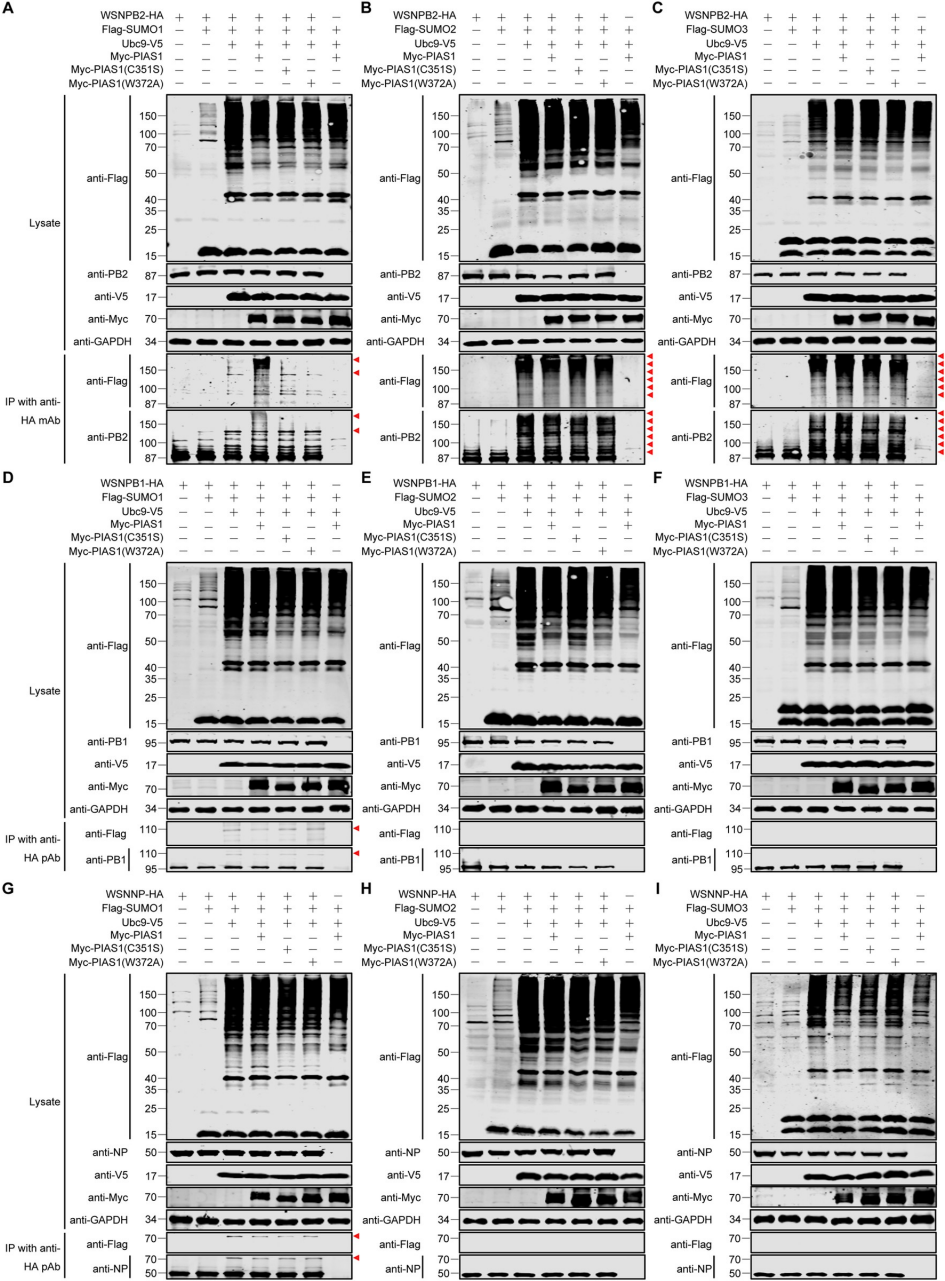
IAV PB2, PB1, and NP are differentially SUMOylated in HEK293T cells expressing exogenous SUMOylation components. (A-I) HEK293T cells were transfected with plasmids expressing HA-tagged WSNPB2 (A-C), WSNPB1 (D-F), or WSNNP (G-I), along with or without Flag-SUMO1/2/3, Ubc9-V5, and Myc-PIAS1 or its mutant. HEK293T cells transfected to express only Flag-SUMO1/2/3, Ubc9-V5, and Myc-PIAS1 served as a negative control. At 36 h post-transfection, cell lysates were immunoprecipitated with a mouse anti-HA mAb (A-C) or a rabbit anti-HA pAb (D-I), and were then subjected to western blotting with a rabbit anti-PB2 pAb (A-C), a mouse anti-PB1 mAb (D-F), a rabbit anti-NP pAb (G-I), and a mouse anti-Flag mAb (A-I) for the detection of PB2, PB1, NP, and SUMO1/2/3, respectively. Red triangle indicates the corresponding SUMOylated viral protein.

PIAS1 interacted with PB2, PB1, and NP of the IAV RNP complex, and suppressed the viral RNP activity through its SUMO E3 ligase activity, which suggests that PIAS1 likely SUMOylates the interacting RNP proteins. To test this possibility, we determined whether PIAS1 could mediate the SUMOylation of IAV PB2, PB1, or NP. First, we examined the SUMOylation of PB2. HEK293T cells were transfected with constructs for the expression of HA-tagged WSNPB2, Flag-tagged SUMO1, SUMO2, or SUMO3, V5-tagged Ubc9, along with Myc-tagged PIAS1, PIAS1-C351S, or PIAS1-W372A. At 36 h post-transfection, cell lysates were immunoprecipitated with an anti-HA mAb, followed by western blotting with an anti-Flag mAb and an anti-PB2 pAb for the detection of SUMOylation. No visible band was produced by the negative control sample from cells expressing only Flag-SUMO1, Ubc9-V5, and Myc-PIAS1 (Fig 5A–5C). A visible SUMOylated band (~125 kDa) above the native bands of PB2 was detected when WSNPB2 was co-expressed with Flag-SUMO1 and Ubc9-V5. Higher molecular weight bands of SUMOylated PB2 (polymeric form, >150 kDa) were observed when WSNPB2 was co-expressed with Flag-SUMO1, Ubc9-V5, and Myc-PIAS1, whereas the PIAS1 C351S and W372A mutants failed to promote poly-SUMOylation of PB2 by SUMO1 (Fig 5A). In the case of PB2 SUMOylation by SUMO2, we found that co-expression of Flag-SUMO2 and Ubc9-V5 efficiently catalyzed the poly-SUMOylation of PB2 by SUMO2, and the further addition of wild-type PIAS1, PIAS1-C351S, or PIAS1-W372A had no additive effect on the SUMOylation of PB2 by SUMO2 (Fig 5B). Regarding the SUMOylation of PB2 by SUMO3, Ubc9 alone catalyzed the poly-SUMOylation of PB2 by SUMO3, and further addition of wild-type PIAS1 slightly enhanced this process, but co-expression of PIAS1-C351S or PIAS1-W372A had no enhancing effect (Fig 5C). Together, these results indicate that with the expression of exogenous Ubc9, PIAS1 is important for the poly-SUMOylation of PB2 by SUMO1, whereas it has no or only a slight effect on the poly-SUMOylation of PB2 by SUMO2 and SUMO3, respectively.

Next, we assessed the SUMO modification of PB1 by PIAS1. PB1 SUMOylation by SUMO1 was not observed when exogenous SUMO1 was expressed alone, but a clear SUMOylated band for PB1 of ~110 kDa was detected when Ubc9 was co-expressed (Fig 5D), which is consistent with the previous report by Li et al [58]. Moreover, the further co-expression of PIAS1, PIAS1-C351S, or PIAS1-W372A produced no additive effect on the SUMOylation of PB1 by SUMO1. Unlike our findings for PB2, the co-expression of Ubc9, or Ubc9 and PIAS1, did not promote the SUMOylation of PB1 by SUMO2 or SUMO3 (Fig 5E and 5F). These data indicate that IAV PB1 is preferentially marked by the SUMO1 chain, and that PIAS1 is not involved in the SUMOylation of PB1 by SUMO1, SUMO2, or SUMO3.

We then examined the SUMOylation of NP by PIAS1. Interestingly, the co-expression of Ubc9 promoted the SUMOylation of NP by SUMO1, but not SUMO2, or SUMO3 (Fig 5G–5I). In addition, the further ectopic expression of PIAS1 led to no further increase in the SUMOylation of NP by SUMO1 (Fig 5G). These results seemed to indicate that the SUMO E3 ligase activity of PIAS1 is not associated with the SUMOylation of NP. To verify these results, we included PIASxα as a control in the SUMOylation experiment because PIASxα has been shown to mediate the SUMOylation of IAV NP [59]. We found that PIASxα interacted with NP in a co-IP experiment (S6A Fig). Interestingly, when Myc-PIASxα was co-expressed with Ubc9-V5, Flag-SUMO1, and WSNNP-HA in HEK293T cells, we did not observe the enhanced effect on the SUMOylation of NP by PIASxα compared with the co-expression of Ubc9-V5 and Flag-SUMO1 (S6B Fig). This result prompted us to speculate that Ubc9 overexpression may mask PIASxα-induced enhancement of NP SUMOylation by SUMO1. To test this hypothesis, WSNNP-HA was co-expressed with Flag-SUMO1 and Myc-PIASxα, and co-expression of WSNNP-HA, Flag-SUMO1, and Ubc9-V5 served as a positive control. As shown in S6C Fig, a similar level of NP SUMOylation by SUMO1 was detected in HEK293T cells overexpressing Myc-PIASxα or Ubc9-V5. To further investigate whether PIAS1 can mediate the SUMOylation of NP by SUMO1 without Ubc9 overexpression, WSNNP-HA and Flag-SUMO1 were co-expressed with Myc-PIAS1, Myc-PIAS1 mutants, or Ubc9-V5 in HEK293T cells. As shown in S6D Fig, only a faint band of SUMOylated NP was observed in PIAS1-overexpressing cells in comparison to the SUMO1-NP band induced by Ubc9 overexpression. Of note, this ~75 kDa band was absent from HEK293T cells transfected with the Myc-PIAS1 C351S or W372A mutant. Taken together, these results indicate that PIAS1 is only minimally involved in the SUMOylation of IAV NP, and that PIASxα is the major SUMO E3 ligase involved in the SUMOylation of NP with SUMO1.

**Fig 6:**
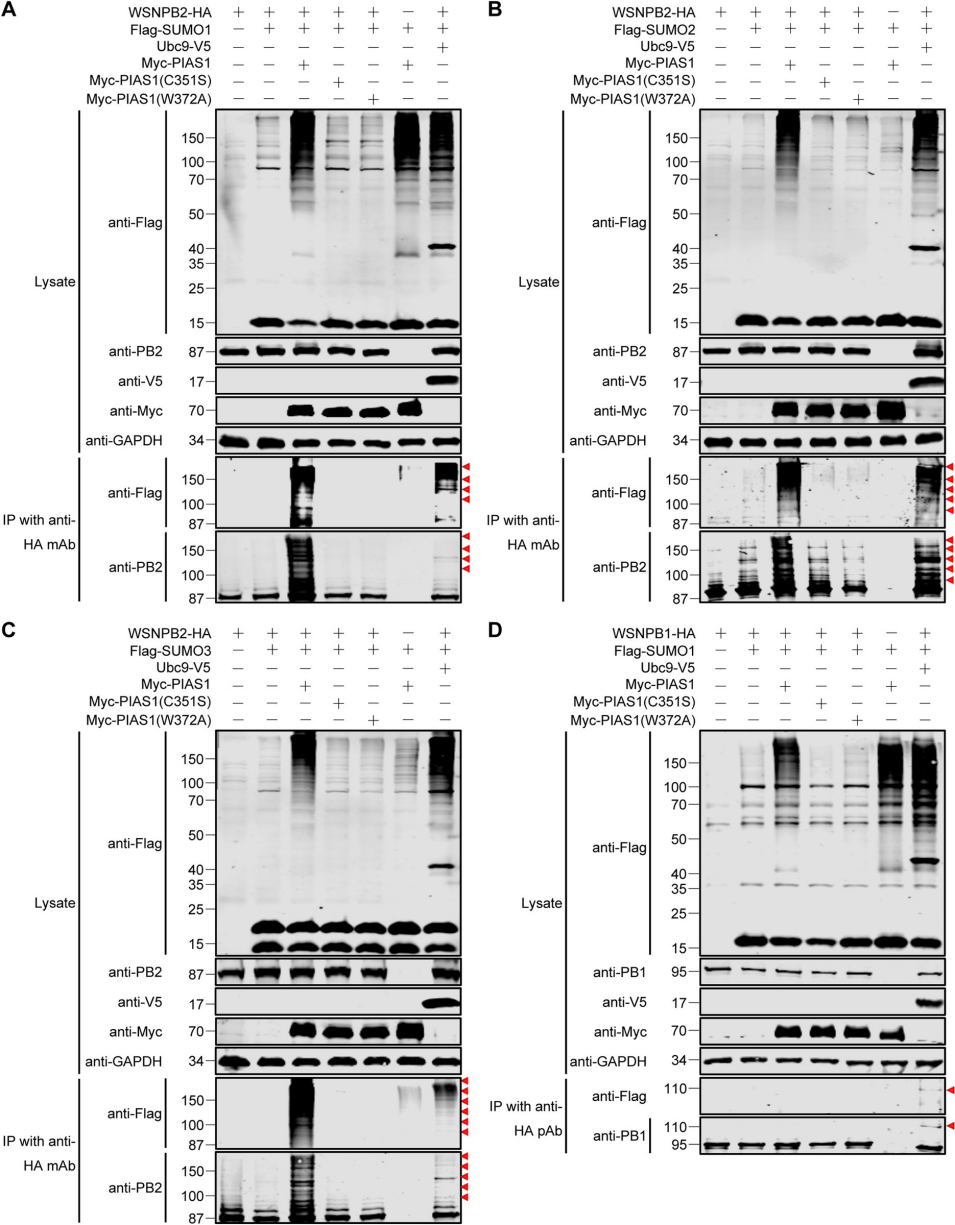
PIAS1 mediates robust SUMOylation of IAV PB2 and has no effect on IAV PB1 SUMOylation. (A-C) PIAS1 catalyzes SUMOylation of IAV PB2 with SUMO1 (A), SUMO2 (B), and SUMO3 (C). HEK293T cells were transfected with plasmids expressing HA-tagged WSNPB2, along with or without Flag-SUMO1 (A), Flag-SUMO2 (B), Flag-SUMO3 (C), Ubc9-V5, Myc-PIAS1, and Myc-PIAS1 mutants. HEK293T cells transfected to express only Flag-SUMO1/2/3 and Myc-PIAS1 served as a negative control. At 36 h post-transfection, cell lysates were immunoprecipitated with a mouse anti-HA mAb, and were then subjected to western blotting with a rabbit anti-PB2 pAb and a mouse anti-Flag mAb for the detection of PB2 and SUMO1 (A), SUMO2 (B), and SUMO3 (C), respectively. (D) PIAS1 has no effect on the SUMOylation of IAV PB1. HEK293T cells were transfected with plasmids expressing HA-tagged WSNPB1, along with or without Flag-SUMO1, Ubc9-V5, Myc-PIAS1, and Myc-PIAS1 mutants. HEK293T cells transfected to express only Flag-SUMO1 and Myc-PIAS1 served as a negative control. At 36 h post-transfection, cell lysates were immunoprecipitated with a rabbit anti-HA pAb, and were then subjected to western blotting with a mouse anti-PB1 mAb and a mouse anti-Flag mAb for the detection of PB1 and SUMO1, respectively. Red triangle indicates the corresponding SUMOylated viral protein.

Given that, in the presence of exogenously expressed Ubc9, a further increase in NP SUMOylation catalyzed by PIAS1 was masked, we investigated whether PIAS1 could mediate the SUMOylation of IAV PB2 and PB1 in the absence of overexpressed Ubc9. To this end, WSNPB2-HA and Flag-SUMO1/2/3 were co-expressed with Myc-PIAS1 or Myc-PIAS1 mutants in HEK293T cells, and co-expression of WSNPB2-HA, Flag-SUMO1/2/3, and Ubc9-V5 served as a positive control. As shown in Fig 6A–6C, smeared poly-SUMOylation bands of PB2 modified by SUMO1, SUMO2, and SUMO3 were easily detected by western blotting with both anti-Flag and anti-PB2 antibodies in the immunoprecipitated samples. The smeared SUMOylation bands modified by SUMO2 and SUMO3 were apparently weaker in the input samples without PB2 expression compared with those that included PB2 (Fig 6B and 6C, see lane 3 and 6), indicating that PIAS1 more specifically mediates the SUMOylation of PB2 by SUMO2 and SUMO3 compared with cellular proteins. With respect to PB1, we further investigated whether PIAS1 is capable of mediating the SUMOylation of PB1 by SUMO1 in the absence of exogenous Ubc9 expression. Interestingly, although Flag-SUMO1 and WSNPB1 were co-expressed with Myc-PIAS1, the SUMOylated form of PB1 was undetectable in the absence of Ubc9 overexpression (Fig 6D), indicating that PIAS1 is unable to mediate the SUMOylation of PB1 by SUMO1.

Collectively, these results clearly demonstrate that the SUMO E3 ligase activity of PIAS1 catalyzes the SUMOylation of PB2 by SUMO1, SUMO2, or SUMO3, but has no role in the SUMOylation of PB1 and plays a minimal role in the SUMOyaltion of NP by SUMO1.

### PIAS1-mediated SUMOylation destabilizes IAV PB2

**Fig 7:**
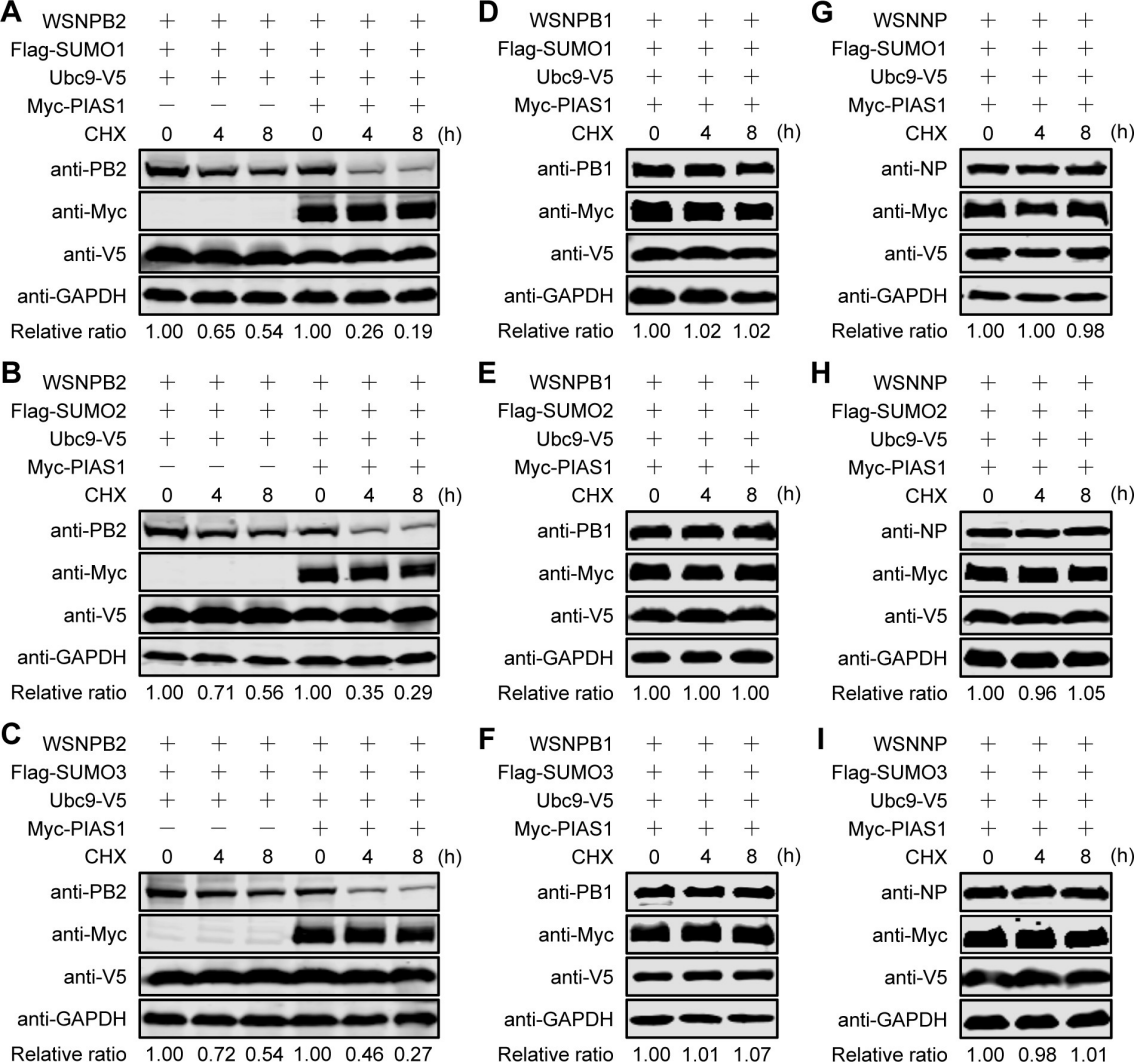
IAV PB2, PB1, and NP exhibit diverse stability in the presence of exogenously expressed SUMOylation components. (A-I) Stability of PB2, PB1 and NP in the presence of exogenous SUMOylation components. HEK293T cells were transfected with plasmids for the expression of WSNPB2, Flag-SUMO1/2/3, Ubc9-V5, along with/without Myc-PIAS1 (A-C), or transfected with plasmids for the expression of WSNPB1 (D-F) or WSNNP (G-I) in combination with Flag-SUMO1/2/3, Ubc9-V5, and Myc-PIAS1. At 36 h post-transfection, the cells were treated with CHX. At the indicated timepoints, cell lysates were subjected to western blotting. Data are representative of three independent experiments (A-I). The band intensities of PB2, PB1, and NP, quantified by using ImageJ software, were normalized to GAPDH and are expressed as relative ratios compared with untreated cells at 0 h.

We showed that PIAS1 differentially promoted the SUMOylation of PB2 and NP, but was not involved in the SUMOylation of PB1. To ascertain the effect of PIAS1-mediated SUMOylation on the viral RNP proteins, we determined their stability upon SUMOylation. PB1 was included as a control even though it was not SUMOylated by PIAS1. First, we examined the effect of SUMO modification by SUMO1, SUMO2, or SUMO3 on the stability of PB2. The constructs expressing WSNPB2, Flag-tagged SUMO1, Ubc9-V5, along with or without Myc-PIAS1, were co-transfected into HEK293T cells. Thirty-six hours later, the cells were treated with cycloheximide (CHX) to inhibit protein synthesis, followed by western blotting analysis at the indicated timepoints. We found that the exogenous expression of SUMO1 and Ubc9 affected the stability of PB2, and the further overexpression of PIAS1 led to dramatically reduced stability of PB2 (Fig 7A), indicating that PIAS1-mediated SUMOylation of PB2 by SUMO1 led to degradation of PB2. Similarly, the stability of PB2 was significantly decreased over time when the Flag-tagged SUMO1 was replaced with SUMO2 or SUMO3 in the transfections (Fig 7B and 7C).

We then evaluated the effect of PIAS1 expression on the stability of PB1. Consistent with the finding that PIAS1 did not catalyze the SUMOylation of PB1 by SUMO1, SUMO2, or SUMO3, the co-expression of SUMO1, SUMO2, or SUMO3, Ubc9, and PIAS1 produced no visible effect on the stability of PB1 (Fig 7D–7F). Similarly, the co-expression of SUMO1, SUMO2, or SUMO3, Ubc9, and PIAS1 did not affect the stability of NP (Fig 7G–7I), implying that PIAS1-mediated minimal SUMOylation of NP by SUMO1 does not cause NP degradation.

**Fig 8:**
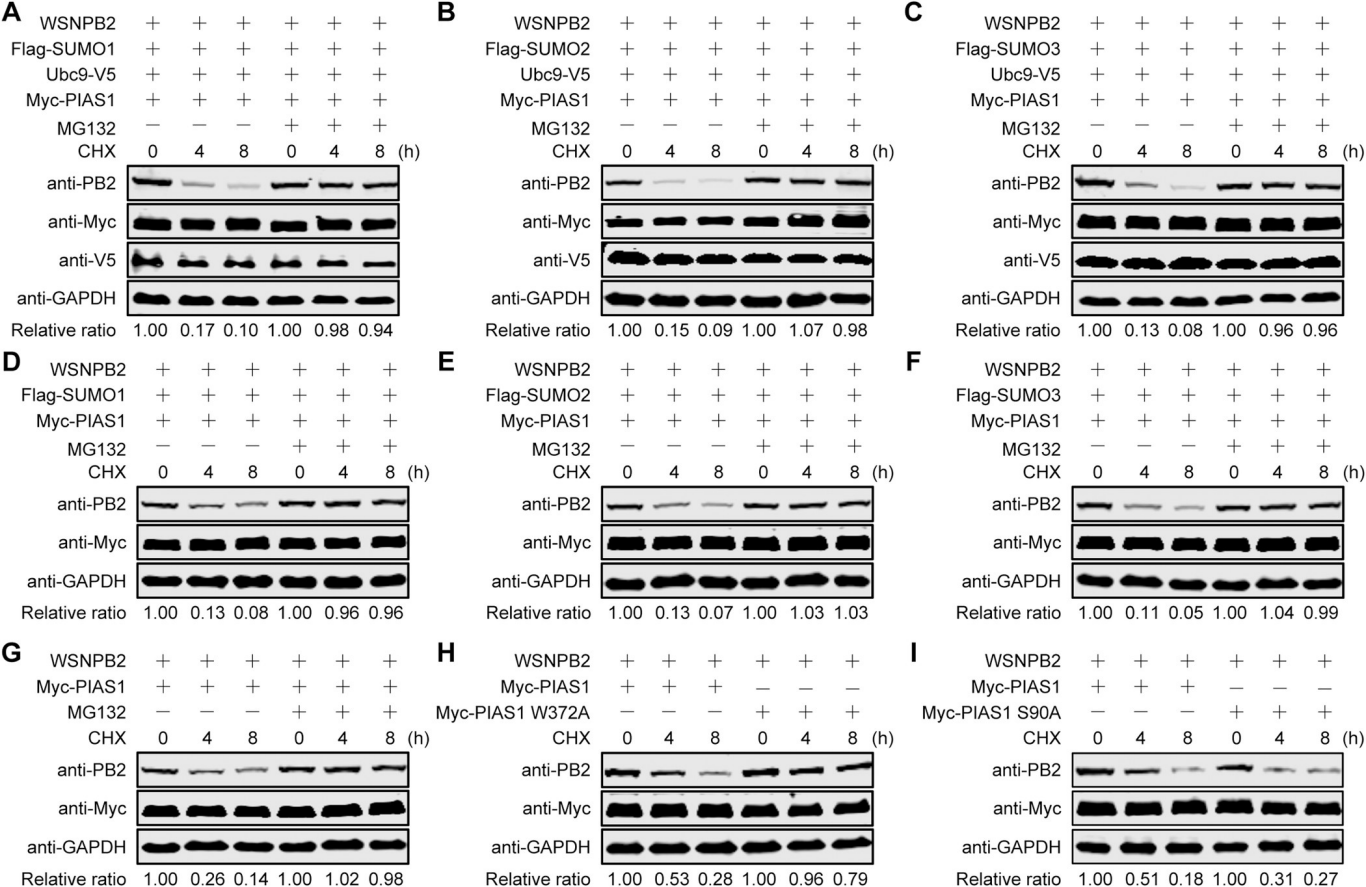
PIAS1-mediated SUMOylation leads to degradation of PB2 through the ubiquitin-proteasome pathway. (A-C) Stability of PB2 in cells overexpressing SUMO1/2/3, Ubc9, and PIAS1 in the presence or absence of MG132. HEK293T cells were transfected with plasmids for the expression of WSNPB2, Ubc9-V5, Myc-PIAS1, and Flag-SUMO1 (A), Flag-SUMO2 (B), or Flag-SUMO3 (C). At 36 h post-transfection, the cells were treated with CHX or with CHX and MG132. At the indicated timepoints, cell lysates were subjected to western blotting. (D-F) Stability of PB2 in cells overexpressing SUMO1/2/3 and PIAS1 in the presence or absence of MG132. HEK293T cells were transfected with plasmids for the expression of WSNPB2, Myc-PIAS1, and Flag-SUMO1 (D), Flag-SUMO2 (E), or Flag-SUMO3 (F). At 36 h post-transfection, the cells were treated with CHX or with CHX and MG132. At the indicated timepoints, cell lysates were subjected to western blotting. (G) Stability of PB2 in cells overexpressing PIAS1 in the presence or absence of MG132. HEK293T cells were transfected with plasmids for the expression of WSNPB2 and Myc-PIAS1. At 36 h post-transfection, the cells were treated with CHX or with CHX and MG132. At the indicated timepoints, cell lysates were subjected to western blotting. (H, I) Effect of the PIAS1 mutant on the stability of PB2. HEK293T cells were transfected with plasmids for the expression of WSNPB2, Myc-PIAS1, and Myc-PIAS1 W372A (H) or Myc-PIAS1 S90A (I). At 36 h post-transfection, the cells were treated with CHX. At the indicated timepoints, cell lysates were subjected to western blotting. Data are representative of three independent experiments (A-I). The band intensities of PB2, quantified by using ImageJ software, were normalized to GAPDH and are expressed as relative ratios compared with untreated cells at 0 h at the bottom of each panel.

To investigate whether SUMOylated PB2 catalyzed by PIAS1 was degraded through the ubiquitin-proteasome pathway, HEK293T cells were co-transfected to express WSNPB2 and exogenous SUMOylation components (Ubc9, PIAS1, and SUMO1, SUMO2, or SUMO3), and were then treated with CHX to inhibit protein synthesis and with the proteasome inhibitor MG132. We found that the co-expression of SUMO1/2/3, Ubc9 and PIAS1 led to clear degradation of PB2, whereas treatment with MG132 prevented PB2 degradation in the presence of overexpressed SUMOylation components (Fig 8A–8C). Consistent with the finding that PIAS1 is able to mediate robust SUMOylation of PB2 by SUMO1/2/3 in the absence of Ubc9 overexpression, the co-expression of only SUMO1/2/3 and PIAS1 led to obvious degradation of PB2, whereas treatment with MG132 subverted the degradation of PB2 (Fig 8D–8F). These results demonstrate that SUMOylated PB2 catalyzed by PIAS1 is degraded through the ubiquitin-proteasome pathway. Furthermore, we found that endogenous SUMO and Ubc9 were sufficient to support PIAS1-mediated degradation of PB2 (Fig 8G), and that the addition of MG132 completely blocked PB2 degradation, causing a similar degradation effect to that observed in the presence of exogenously expressed SUMO and Ubc9.

To confirm that PIAS1-mediated SUMOylation was responsible for the reduced stability of PB2, we did a side-by-side comparison of the effect of wild-type PIAS1 versus the PIAS1 S90A or W372A mutant on the stability of PB2. We found that the presence of the SUMO-ligase defective PIAS1 W372A mutant prevented the reduction in stability of PB2 (Fig 8H). In contrast, the SUMO-ligase active PIAS1 S90A mutant dramatically impaired the stability of PB2, demonstrating that PIAS1 is dependent on its SUMO-ligase activity to reduce the stability of PB2 (Fig 8I).

Collectively, these results demonstrate that PIAS1-mediated SUMOylation of PB2 by SUMO1, SUMO2, and SUMO3 dramatically decreases the stability of PB2, but PIAS1-mediated SUMOylation of NP by SUMO1 has no effect on the stability of NP.

### Pias1 suppresses the virulence of IAV in mice

**Fig 9:**
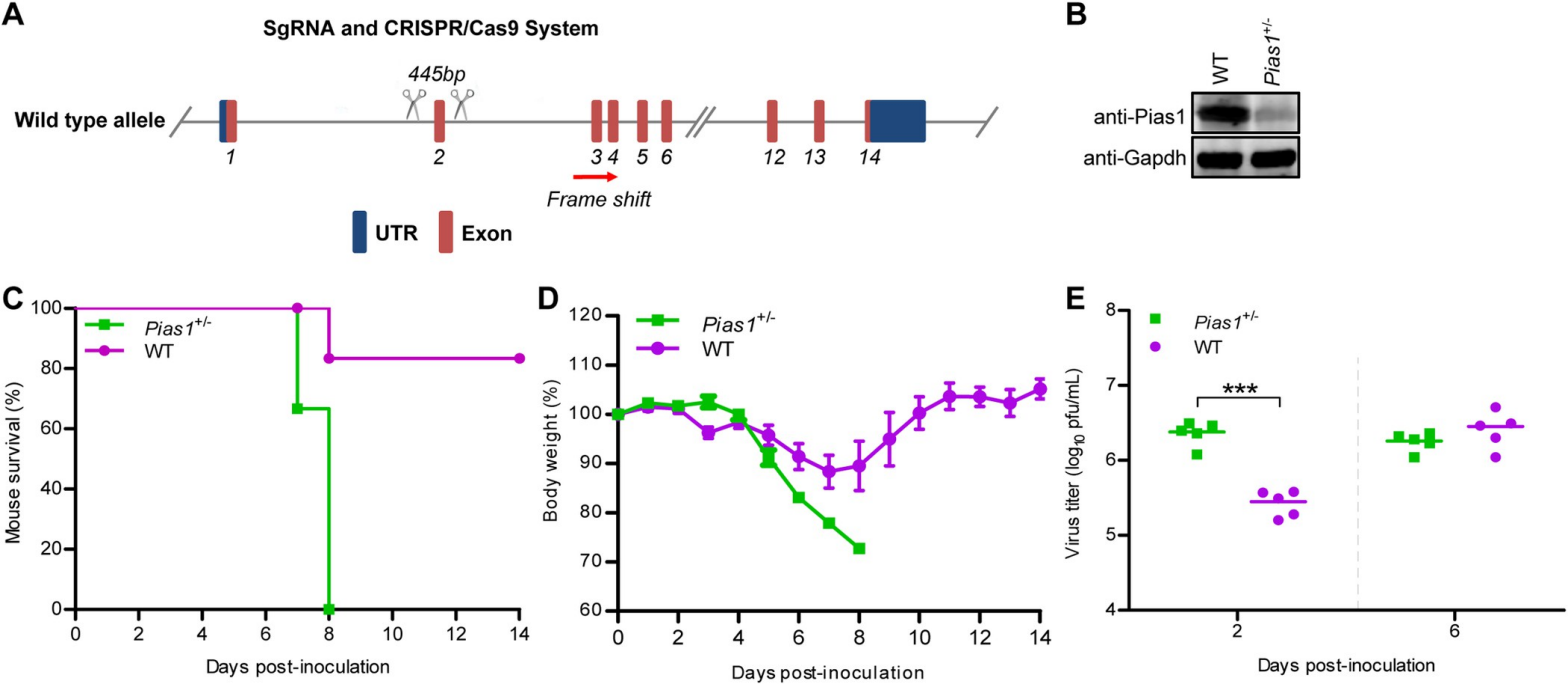
Pias1 inhibits IAV replication and virulence in mice. (A) Schematic illustration of the strategy used to generate *Pias1*^+/-^ mice. Two sgRNAs were designed to delete exon 2 of the *Pias1* gene. (B) The genotype of *Pias1*^+/-^ mice was verified by western blotting with a rabbit anti-PIAS1 pAb. (C) Survival of WT and *Pias1*^+/-^ mice (n = 6 per group) after intranasal infection with WSN (H1N1) virus (1.2×10^4^ PFU/mouse). (D) Body weight changes of WT and *Pias1*^+/-^ mice (n = 6 per group) after intranasal infection with WSN (H1N1) virus. (E) Titers of WSN (H1N1) virus in the lungs of WT and *Pias1*^+/-^ mice (n = 5 per group) on days 2 and 6 p.i.

To investigate the role of PIAS1 in the replication and virulence of IAV in vivo, we attempted to generate *Pias1* knockout (*Pias1*_KO) mice by using the CRISPR/Cas9 system. The targeting construct was designed to delete exon 2 of the mouse *Pias1* gene (Fig 9A). Homozygous *Pias1*_KO mice could not be generated, reflecting the importance of this gene in vivo. However, we successfully generated heterozygous *Pias1*_KO (*Pias1*^*+/-*^) mice, which were normal in size and did not display any gross physical or behavioral abnormalities. The expression of Pias1 in the lung extracts of *Pias1*^*+/-*^ mice was dramatically decreased compared with that in wild-type mice (Fig 9B). *Pias1*^*+/-*^ and wild-type mice were intranasally inoculated with 1.2×10^4^ PFU of WSN (H1N1) virus, and their body weight loss and mortality were monitored daily for 14 days. We found that all six *Pias1*^*+/-*^ mice died of their infection on day 7 or 8, whereas 5 out of 6 wild-type mice survived (Fig 9C). In addition, infected *Pias1*^*+/-*^ mice continued to lose body weight until death (Fig 9D). By contrast, the wild-type mice that survived gained body weight at the late stage of infection (Fig 9D). The virus titers in lung homogenates of infected mice were determined on days 2 and 6 p.i. We found that the viral titers in the lungs of *Pias1*^*+/-*^ mice on day 2 p.i. were significantly higher than those of wild-type mice, whereas no significant difference was observed on day 6 p.i. (Fig 9E). These results indicate that the reduction in Pias1 expression in the *Pias1*^*+/-*^ mice led to enhanced virus replication at early time points of infection and increased virus pathogenicity.

## Discussion

PIAS1 has been reported to modulate the life cycle of different viruses, such as EBV, EBOV, HSV-1, and VSV [29,50–53], playing roles as both an enhancer and inhibitor of virus replication. Here, we found that PIAS1 negatively regulates the replication of IAV. SiRNA knockdown or CRISPR/Cas9 knockout of PIAS1 expression dramatically increased the replication of IAV, whereas lentiviral-mediated overexpression of PIAS1 led to markedly reduced propagation of IAV. Consistent with these findings, we found that PIAS1 expression suppressed viral RNP complex activity. Importantly, we found that reduced expression of PIAS1 markedly enhanced the replication and virulence of IAV in *Pias1*^*+/-*^ mice compared with wild-type mice. Taken together, these data indicate PIAS1 is a critical restriction factor for the replication of IAV in vitro and in vivo.

A broad range of cellular events involve the engagement of PIAS1, whose roles are executed in a variety of ways [47]. Primarily, PIAS1-mediated SUMOylation of target proteins alters their function, localization, or interaction with other partners [47]. In the present study, we found that the expression of PIAS1 significantly reduced the RNP complex activity of IAV. The generation of PIAS1 mutants showed that the phosphorylation mutant PIAS1-S90A retained the ability to reduce the viral RNP complex activity. By contrast, the C351S or W372A mutation, which abolish the SUMO E3 ligase activity of PIAS1 [30,57], prevented PIAS1-mediated suppression of viral RNP complex activity. These results indicate that the SUMO E3 ligase activity is essential for PIAS1 to suppress the RNP complex activity of IAV.

The PIAS1 protein was initially identified as an interacting partner of viral NP protein by using the yeast two-hybrid assay. Interestingly, PIAS1 could interact independently with three of the four protein components of the RNP complex of IAV. Further investigation showed that PIAS1 mediates robust SUMOylation of PB2 and minimal SUMOylation of NP. Previous studies have demonstrated that IAV proteins are targets of the host SUMOylation system [60], and that IAV infection triggers a global increase in the cellular SUMOylation response [56]. From these studies, it appears that SUMOylation of PB1 is important for its ability to bind to viral RNA and for viral pathogenesis and transmission [58], SUMOylation of NP is essential for the intracellular trafficking of NP and virus growth [59], SUMOylation of M1 promotes the assembly and morphogenesis of virus particles [61], and SUMOylation of NS1 enhances its stability [62], modulates its interferon-blocking activity [63], or suppresses host gene expression by inducing global RNAPII termination defects [64]. Together, these previous studies showed that the SUMOylation of these four IAV proteins is beneficial to the virus replication cycle. Similarly, SUMOylation of the proteins of other viruses, for example, NS5 of dengue virus, 3D protein of enterovirus 71, and VP1 of avibirnavirus, is also beneficial for the replication of the corresponding virus [65–67]. However, there are also some exceptions: the SUMOylation of the phosphoprotein of human parainfluenza virus type 3 impairs virus replication [68], and PIAS1-mediated SUMOylation promotes enterovirus 3C protein degradation and causes reduced virus replication [69]. Here, we also demonstrated that PIAS1-mediated SUMOylation of PB2 reduced its stability, thereby suppressing viral RNP complex activity and virus replication. Consistent with the findings of Domingues et al [56], our results showed that IAV infection induces a global increase in cellular SUMOylation. Moreover, we found that knockdown of PIAS1 expression reduces the overall level of cellular SUMOylation during IAV infection. In addition to the major antiviral role of PIAS1 in mediating SUMOylation and degradation of PB2, we cannot exclude the possibility that PIAS1 also indirectly affects IAV infection by modulating the SUMOylation of cellular proteins.

PB2 is important for the RNP complex to catalyze the transcription and replication of the viral genome, and plays a pivotal role in mammalian adaptation, pathogenesis, and transmission of IAV [4,70–76]. We recently identified PB2 as a target of TRIM35, which induces the K48-linked ubiquitination and proteasomal degradation of this viral protein [20]. Here we found that PIAS1 catalyzes the SUMOylation of PB2 with SUMO1, SUMO2, and SUMO3. Notably, the SUMOylation of PB2 with SUMO1, SUMO2, or SUMO3 all resulted in reduced stability of PB2, and treatment with the proteasome inhibitor MG132 completely inhibited the degradation of PB2. These results indicate that PIAS1-mediated poly-SUMOylated PB2 is degraded via the ubiquitin-proteasome pathway. These findings mirror a previous report in which PIAS1 mediates the ligation of multiple SUMO2/3 monomers to c-Myc, which is subsequently ubiquitinated by RNF4 and degraded by proteasomes [77]. Generally, unlike SUMO2 and SUMO3, SUMO1 is unable to be conjugated as polymeric chains to a target protein due to the lack of a lysine residue in a consensus site [78]. In contrast, SUMO1 can serve as a terminator of chain elongation upon its attachment to residue K11 of the distal SUMO2/3 moiety [79]. Given that PB2 can be poly-SUMOylated by SUMO2 and SUMO3, it is highly likely that the smeared poly-SUMOylation bands of PB2 catalyzed by PIAS1 with SUMO1 were hybrid chains created by the distal attachment of SUMO1 to the SUMO2/3 chains of the poly-SUMOylated PB2. Since SUMO can act as a signal for the recruitment of E3 ubiquitin ligases [80], our findings suggest that the SUMOylation of PB2 most likely recruits SUMO-targeted ubiquitin ligases (STUbLs), such as RNF4 and RNF111 [77,81], to the SUMOylated PB2, resulting in ubiquitination-dependent proteasomal degradation of PB2. However, the identity of such STUbLs involved in the proteasomal degradation of PB2 remains to be investigated.

In contrast, PIAS1 only catalyzed minimal SUMOylation of NP by SUMO1, and yielded no visible effect on the stability of NP. Moreover, PIAS1 did not promote the SUMOylation of PB1 with any of the SUMO molecules. Although PB2, PB1, and NP are integral components of the vRNP complex and all of them can interact with PIAS1, PIAS1 only catalyzed the robust SUMOylation of PB2 by SUMO1, SUMO2, and SUMO3, which may be influenced by the structural characteristics of PB2. In brief, our data indicate that the interaction between PIAS1 and RNP complex proteins might be a complex and dynamic process, and SUMOylation by the same SUMO E3 ligase can produce different effects due to differences in the substrates, even when they belong to the same functional complex.

There are fewer than ten bona fide SUMO E3 ligases in humans: the SP-RING family (PIAS1-4, Nse2), RanBP2, and the ZNF451 family [82]. We found that PIAS1 not only interacts with IAV PB2, but also SUMOylates it by SUMO1, SUMO2, and SUMO3. Interestingly, PIAS1 did not catalyze the SUMOylation of PB1 even though PIAS1 and PB1 interacted with each other. Moreover, although PIAS1 catalyzed the SUMOylation of NP with SUMO1, the modification intensity was apparently weaker than that by PIASxα. These results indicate that the SUMOylation preference differs with the identity of the SUMO E3 ligase. Whether other SUMO E3 ligases could also catalyze the SUMOylation of the IAV RNP complex proteins remains to be investigated.

**Fig 10:**
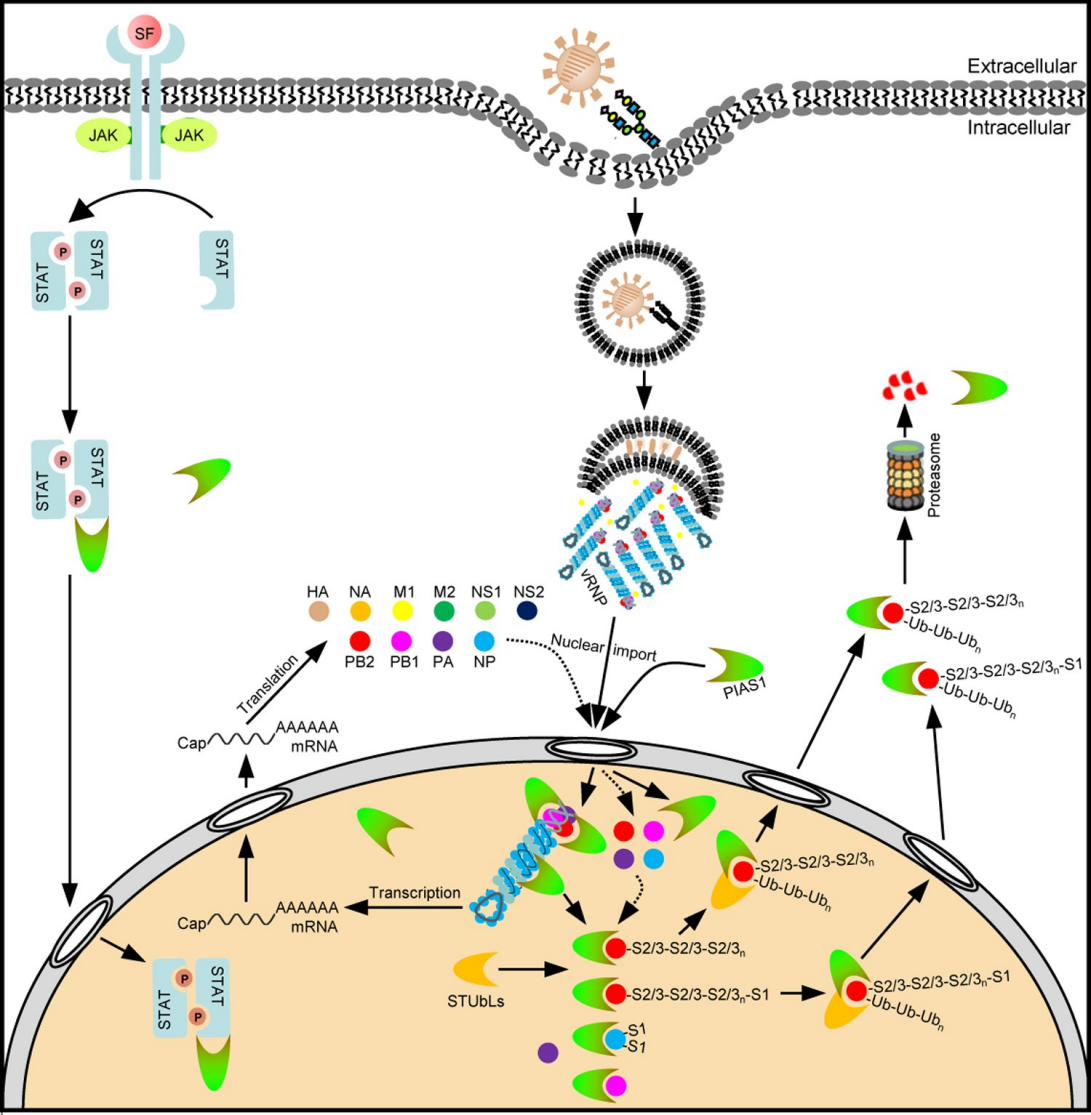
Schematic model of PIAS1-mediated SUMOylation and degradation of IAV PB2. Upon initial binding with sialic acid receptors on the cell surface, IAV is endocytosed into host cells. Following low-pH mediated fusion of viral and endosomal membranes, the vRNPs are released into the cytoplasm, and translocate into the nucleus where they catalyze the transcription and replication of viral genome. PIAS1 mediates robust poly-SUMOylation of PB2 from incoming vRNPs or newly synthesized PB2 by SUMO1/2/3, and also catalyzes minimal SUMOylation of NP by SUMO1. PIAS1-mediated SUMOylation of PB2 most likely recruits SUMO-targeted ubiquitin ligases (STUbLs) to the SUMOylated PB2, resulting in ubiquitination-dependent proteasomal degradation of PB2. SF: stimulating factors.

In summary, here we demonstrate that PIAS1 interacts with PB2, PB1, and NP of the IAV RNP complex. The SUMO E3 ligase activity is essential for PIAS1 to suppress the viral RNP complex activity. Mechanistically, PIAS1-mediated SUMOylation of viral PB2 protein dramatically reduces its stability (Fig 10). The expression of PIAS1 is markedly induced upon IAV infection, which appears to restrict virus replication in vitro and suppress virulence in mice. Manipulation of this host defense mechanism may improve our ability to control IAV infections.

## Materials and methods

### Ethics statements

This study was carried out in strict accordance with the recommendations in the Guide for the Care and Use of Laboratory Animals of the Ministry of Science and Technology of the People’s Republic of China. The protocols for mouse studies were approved by the Committee on the Ethics of Animal Experiments of the Harbin Veterinary Research Institute (HVRI) of the Chinese Academy of Agricultural Sciences (CAAS) (approval number BRDW-XBS–19).

### Facility

All experiments with A/Anhui/2/2005 (AH05, H5N1) virus and A/Anhui/1/2013 (AH13, H7N9) virus were conducted within the enhanced animal biosafety level 3 (ABSL3+) facility in the HVRI, CAAS, which is approved for such use by the Ministry of Agriculture and Rural Affairs of China and the China National Accreditation Service for Conformity Assessment. The details of the facility and the biosafety and biosecurity measures used have been previously reported [83].

### Cells and viruses

Human embryonic kidney cells (HEK293T), human lung carcinoma cells (A549), and Madin-Darby canine kidney (MDCK) cells were cultured in DMEM (Life Technologies, Grand Island, NY, USA) supplemented with 10% fetal bovine serum (FBS, Sigma-Aldrich, St. Louis, MO, USA), F-12K medium (Life Technologies) supplemented with 10% FBS, and DMEM containing 6% newborn calf serum (NCS, Sigma-Aldrich), respectively. All media contained 100 units/mL penicillin and 100 μg/mL streptomycin (Life Technologies). All cells were maintained at 37°C in a 5% CO_2_ humidified incubator.

A/chicken/Shanghai/SC197/2013 (SH13, H9N2) virus was grown in 10-day-old embryonated chicken eggs [84]. AH05 (H5N1), AH13 (H7N9), and A/WSN/33 (WSN, H1N1) were propagated in embryonated chicken eggs or MDCK cells as previously described [19].

### Yeast two-hybrid assay

The yeast two-hybrid screen to identify potential binding partners of NP was performed by using the match maker yeast two-hybrid system (Clontech, Mountain View, CA, USA) as previously described [19]. Briefly, NP of AH05 (H5N1) was constructed in pGBKT7 to fuse with the GAL4-binding domain (BD), and used as bait. cDNAs prepared from a mixed human cell culture comprising A549, HEK293T, THP-1, and U251 were cloned into pGADT7 to fuse with the GAL4-activation domain (AD), and used as prey. The yeast strain Y2HGold was transformed with the pGBKT7-AH05NP bait, and was then mated with the Y187 strain transformed with the pGADT7-based cDNA library. Transformants were selected on plates with synthetically defined medium lacking adenine, histidine, leucine, and tryptophan (SD/–Ade/–His/–Leu/–Trp) (quadruple dropout medium, QDO). The recovered colonies were grown on QDO plates containing 5-bromo-4-chloro-3-indolyl-α-d-galactopyranoside (X-α-Gal) and aureobasidin A (AbA) (SD/–Ade/–His/–Leu/–Trp/X-a-Gal/AbA, QDO/X/A). Blue colonies were cultured in medium lacking leucine and tryptophan (SD/−Leu/−Trp) (double dropout medium, DDO). Plasmids were sequenced to identify the potential cellular interactants of NP. One of the potential binding partners of NP identified was PIAS1. To demonstrate the interaction of PIAS1 and NP in yeast, the bait plasmid pGBKT7-AH05NP and prey plasmid pGADT7-PIAS1 were co-transformed into the Y2HGold strain. Co-transformation of pGADT7-T (AD-T) with pGBKT7-p53 (BD-p53) into Y2HGold was used as a positive control, and co-transformation of AD-T with pGBKT7-Lamin (BD-Lam) served as a negative control.

### Plasmids

The *PIAS1*, *PIASxα*, *Ubc9*, *SUMO1*, *SUMO2*, and *SUMO3* genes were amplified by RT-PCR from total cellular mRNAs of A549 cells. Wild-type *PIAS1*, *PIAS1* mutants (S90A, C351S, and W372A), and *PIASxα* were cloned into the mammalian expression vector pCAGGS with a Myc tag at the C-terminus, *Ubc9* was cloned into pCAGGS with a V5 tag at the C-terminus, and *SUMO1*, *SUMO2*, *and SUMO3* were cloned into pCAGGS with a Flag tag at the N-terminus. *PIAS1* was also cloned into the PET-32a(+) vector containing a His Tag (Novagen, Madison, WI, USA), and the pLVX-IRES-ZsGreen1 vector (Clontech). pHH21-SC09NS F-Luc, used to produce an influenza vRNA-like luciferase reporter, has been previously described [19]. Open reading frames (ORFs) of *PB2*, *PB1*, *PA*, and *NP* of WSN (H1N1) virus were cloned into pCAGGS. ORFs of *PB2*, *PB1*, and *NP* of WSN (H1N1) virus were also cloned into pCAGGS with a HA tag at the C-terminus. The ORF of the *NP* of WSN (H1N1) virus was also cloned into pCAGGS with a V5 or GST tag at the N-terminus. All of the constructs used in this study were verified by sequencing.

### Antibodies

The commercially obtained primary antibodies used in this study were: mouse anti-Flag monoclonal antibody (mAb) (A00187-100, GenScript, Nanjing, China), rabbit anti-Flag polyclonal antibody (pAb) (A00170-40, GenScript), mouse anti-HA mAb (A01244-100, GenScript), rabbit anti-HA pAb (51064-2-AP, Proteintech, Wuhan, China), mouse anti-Myc mAb (A00704-100, GenScript), rabbit anti-Myc pAb (A00172-40, GenScript), mouse anti-V5 mAb (A01724-100, GenScript), rabbit anti-V5 pAb (AB3792, Sigma-Aldrich, St. Louis, MO, USA), rabbit anti-GAPDH pAb (10494-1-AP, Proteintech), rabbit anti-MX1 pAb (13750-1-AP, Proteintech), rabbit anti-IFITM3 pAb (GTX63349, GeneTex, Irvine, CA, USA), rabbit anti-PIAS1 pAb (23395-1-AP, Proteintech), rabbit anti-PIAS1 pAb (ab109388, Abcam, Cambridge, MA, USA), rabbit anti-SUMO1 pAb (ab32058, Abcam), rabbit anti-SUMO2/3 mAb (4971, Cell Signaling Technology, Danvers, MA, USA), rabbit anti-HA (IAV) pAb (11692-T54, Sino Biological, Beijing, China), rabbit anti-NA pAb (GTX629696, GeneTex), rabbit anti-M1 pAb (GTX125928, GeneTex), rabbit anti-M2 pAb (GTX125951, GeneTex), and rabbit anti-PB2 pAb (GTX125926, GeneTex). The mouse anti-PB2 mAb, mouse anti-PB1 mAb, rabbit anti-PB1 pAb, mouse anti-PA mAb, rabbit anti-PA pAb, mouse anti-NP mAb and rabbit anti-NP pAb were made and stored in our laboratory. Alexa Fluor 488 goat anti-rabbit IgG (H+L) (A11034) and Alexa Fluor 633 goat anti-mouse IgG (H+L) (A21052), obtained from Life Technologies, were used as secondary antibodies for confocal microscopy. DyLight 680 goat anti-rabbit IgG (H+L) (RS23720), DyLight 800 goat anti-rabbit IgG (H+L) (RS23920), DyLight 680 goat anti-mouse IgG (H+L) (RS23710) and DyLight 800 goat anti-mouse IgG (H+L) (RS23910), obtained from Immunoway (Plano, TX, USA), were used as secondary antibodies for western blotting.

### Western blotting and co-immunoprecipitation (co-IP) analysis

HEK293T cells grown in 6-well plates were transfected with the indicated plasmids by using the Lipofectamine LTX and Plus Reagents (Invitrogen, Carlsbad, CA, USA), or were subsequently infected with WSN (H1N1) virus (MOI = 5). Cell lysates were prepared at 36 h post-transfection or 30 h post-infection. Briefly, the cells were lysed with IP buffer (25 mM Tris-HCl pH 7.4, 150 mM NaCl, 1% NP-40, 1 mM EDTA, 5% glycerol; Pierce, Rockford, IL, USA) containing complete protease inhibitor cocktail (Roche Diagnostics GmbH, Mannheim, Germany) for 30 min on ice, and then centrifuged at 12,000 rpm at 4°C for 20 min. Supernatants were then used as input for western blotting or were subjected to further co-IP assays. For the co-IP analysis, the supernatants were mixed with the respective primary antibodies, rocked at 4°C for 6 h, mixed with Protein G-Agarose beads (Roche) and then rocked for a further 6 h. The beads were washed 4–6 times with wash buffer (25 mM Tris-HCl pH 7.4, 150 mM NaCl, 1 mM PMSF).

Supernatants of cell lysates or immunoprecipitates from the co-IP assays were separated by sodium dodecyl sulfate-polyacrylamide gel electrophoresis (SDS-PAGE), and transferred onto nitrocellulose membranes (GE Healthcare, Pittsburgh, PA, USA). Membranes were blocked with 5% skim milk in PBS, and incubated overnight at 4°C with the appropriately diluted primary antibody in PBS containing 0.5% BSA. After incubation with goat anti-rabbit IgG(H+L) and goat anti-mouse IgG(H+L) secondary antibodies, membranes were scanned by using an Odyssey CLX infrared imaging system (Li-Cor BioSciences, Lincoln, NE, USA). Images were acquired, cropped into suitable size, and saved as TIF format by using an Image Studio software. The individual cropped TIF images were assembled into panels in Powerpoint, and then the whole figure was saved as a PDF file, which was converted into a final TIF image by using Photoshop.

### GST pull down

His-tagged PIAS1 protein was expressed in *E*. *coli* BL21 (DE3) and purified by using Ni Sepharose Excel resin (GE Healthcare). HEK293T cells grown in 6-cm dishes were individually transfected with 5 μg of pCAGGS-GST or pCAGGS-GST-NP plasmid by using the Lipofectamine LTX and Plus Reagents. At 48 h post-transfection, cells were lysed with IP buffer containing a complete protease inhibitor cocktail for 30 min on ice at 4°C and then centrifuged at 12,000 rpm for 20 min at 4°C. The supernatants were mixed with Glutathione Sepharose 4 Fast Flow, rocked for 4 h at 4°C, and washed three times with ice-cold PBS. An equal amount of purified His-tagged PIAS1 was mixed with the Glutathione Sepharose 4 Fast Flow samples that bound GST or GST-NP. The mixtures were rocked for 4 h at 4°C, washed three times with ice-cold PBS, separated by SDS-PAGE, and then Coomassie blue stained by using QuickBlue fast staining solution (Beijing Biodragon Immunotechnologies, Beijing, China).

### SiRNA knockdown and virus replication in A549 cells

A549 cells in 12-well plates were transfected with 20 nM *PIAS1* siRNA (5’-GUGCGGAACUAAAGCAAAU-3’) or scrambled siRNA (5’-UUCUUCGAACGUGUCACGU-3’) (GenePharma, Shanghai, China) diluted in Opti-MEM (Life Technologies) by using the RNAiMAX reagent (Invitrogen). At 48 h post-transfection, PIAS1 knockdown was confirmed by quantitative reverse transcription PCR (RT-qPCR) or western blotting with a rabbit anti-PIAS1 pAb. The siRNA-treated A549 cells were infected with WSN (H1N1) (MOI = 0.01), AH05 (H5N1) (MOI = 0.1), AH13 (H7N9) (MOI = 0.1) or SH13 (H9N2) (MOI = 0.1) virus. Supernatant samples (three replicates for each group) were collected at 24 and 48 h p.i., and virus titers were determined by means of plaque assays on MDCK cells.

### Establishment of a *PIAS1*_KO A549 or HEK293T cell line and virus infection

To generate *PIAS1*_KO A549 cells or *PIAS1*_KO HEK293T cells, the single guide RNA (sgRNA) targeting the *PIAS1* gene was designed by using an online CRISPR Design tool (Dr. Feng Zhang’s lab website). The pSpCas9(BB)-2A-GFP (pX458) vector containing an expression cassette of Cas9 and EGFP was digested by using FastDigest BpiI (Thermo Fisher Scientific, Waltham, MA, USA), and then the sgRNA sequence, 5’-CAAGTACTGTTGGGCTACGC-3’ or 5’-GAACATGTAAGGGCCCGACA-3’, was cloned into the digested pX458 vector. The two pX458 constructs (2 μg each) were simultaneously electrotransfected into A549 cells by using the Neon Transfection System (Thermo Fisher Scientific) or transfected into HEK293T cells by using Lipofectamine LTX and Plus Reagents. Forty-eight hours later, the transfected cells were trypsinized, and the single EGFP-positive cells were seeded into each well of a 96-well plate for colony formation by using a MoFlo XDP cell sorter (Beckman Coulter, Brea, CA, USA). Each colony was propagated in 24-well plates, and the loss of PIAS1 expression was confirmed by western blotting with a rabbit anti-PIAS1 pAb.

The *PIAS1*_KO A549 cells or control A549 cells were infected with WSN (H1N1) virus (MOI = 0.01). Supernatant samples (three replicates for each group) were collected at 24 and 48 h p.i., and virus titers were determined by means of plaque assays on MDCK cells.

### Generation of stable PIAS1-overexpressing A549 cells and virus infection

HEK293T cells grown in 10-cm dishes were transfected with pLVX-IRES-ZsGreen1-PIAS1, psPAX2, and pMD2.G (4:3:1) by using Lipofectamine LTX and Plus Reagents. At 48 h post-transfection, viral supernatants were collected and used to transduce A549 cells in 6-well plates. Forty-eight hours later, EGFP-positive A549 cells were harvested by using a MoFlo XDP cell sorter (Beckman Coulter), and were cultured in 6-well plates. PIAS1 overexpression was confirmed by western blotting with a rabbit anti-PIAS1 pAb. The PIAS1-overexpressing or control A549 cells were infected with WSN (H1N1) virus (MOI = 0.01). Supernatant samples (three replicates for each group) were collected at 24 and 48 h p.i., and virus titers were determined by means of plaque assays on MDCK cells.

### Cell viability

Cell viability was determined by using a CellTiter-Glo luminescent cell viability assay (Promega, Madison, WI, USA) as described previously [85]. Briefly, siRNA-treated A549 cells, *PIAS1*_KO A549 cells or relative control A549 cells were grown in opaque-walled 96-well plates to 95% confluence. Then, 100 μl of CellTiter-Glo reagent was added directly into each well to lyse the cells on a shaker for 10 min, and the luminescence was measured with a GloMax 96 Microplate Luminometer (Promega).

### Dual-luciferase reporter assay

HEK293T cells in 12-well plates were transfected with 20 nM *PIAS1* siRNA or scrambled siRNA diluted in Opti-MEM by using the RNAiMAX reagent. At 12 h post-transfection, the siRNA-treated HEK293T cells were transfected with four viral RNP complex protein plasmids (WSNPB2, WSNPB1, WSNPA, and WSNNP), pHH21-SC09NS F-Luc, and pRL-TK. Thirty-six hours later, the luciferase assay (three replicates for each group) was performed by using the dual luciferase reporter assay system (Promega), and the luciferase activities were measured on a GloMax 96 Microplate Luminometer. Data were normalized for transfection efficiency by the relative ratio between the firefly luciferase value and the internal control Renilla luciferase value.

In a separate experiment, HEK293T cells or *PIAS1*_KO HEK293T cells in 12-well plates were transfected with four viral RNP complex protein plasmids (WSNPB2, WSNPB1, WSNPA, and WSNNP), pHH21-SC09NS F-Luc, and pRL-TK, together with a gradient increase of plasmids expressing wild-type Myc-PIAS1, or S90A, C351S, or W372A mutant of Myc-PIAS1 by using Lipofectamine LTX and Plus Reagents. When applicable, empty pCAGGS was used to adjust for the difference in the amount of transfected plasmids among different wells. At 36 h post-transfection, the luciferase assay (three replicates for each group) was performed as described above.

### PIAS1 expression in A549 cells and mice during IAV infection

A549 cells grown in 12-well plates were infected with WSN (H1N1) virus (MOI = 0.1). Whole cell lysates were prepared with SDS-PAGE loading buffer (Solarbio Science & Technology, Beijing, China) at 0, 12, and 24 h p.i. For the in vivo experiment, C57BL/6J mice were lightly anesthetized with CO_2_, and inoculated intranasally with 10^5^ PFU of WSN (H1N1), 10^2^ PFU of AH05 (H5N1), 10^5^ PFU of AH13 (H7N9), or 10^6^ PFU of SH13 (H9N2) virus. The mice were euthanized on day 3 p.i., and lungs were collected to be homogenized and lysed with SDS-PAGE loading buffer. The protein samples were separated by SDS-PAGE, transferred onto nitrocellulose membranes, and detected by western blotting with a rabbit anti-PIAS1 pAb.

### RNA quantification

Total RNA in *PIAS1* siRNA-treated A549 cells or virus-infected A549 cells (three replicates for each group) was extracted by using the RNeasy Plus Mini Kit (QIAGEN, Valencia, CA, USA) or TRIzol reagent (Life Technologies) according to the manufacturers’ instructions. The first-strand cDNAs were synthesized with oligo (dT) primer and random 6 mers or *NP-*specific primers by using the PrimeScript RT reagent Kit with gDNA Eraser (TaKaRa, Dalian, China) [86]. RT-qPCR assays were performed by using SYBR premix Ex Taq II (TaKaRa). Relative RNA quantities were determined by using the comparative cycle-threshold method, with cellular *GAPDH* serving as the internal control. Dissociation curve analysis was performed after each assay to ensure specific detection.

### Confocal microscopy

To observe the localization of NP and PIAS1 during IAV infection, A549 cells were grown in glass-bottom dishes to 90% confluence, and then infected with WSN (H1N1) virus (MOI = 5). At 2, 4, 6, and 8 h p.i., cells were fixed with 4% paraformaldehyde (PFA, Solarbio Science & Technology) for 20 min at room temperature, and permeabilized with 0.5% Triton X-100 in PBS for 20 min. The permeabilized cells were blocked with 5% BSA in PBS for 1 h, and then incubated with a mouse anti-NP mAb (1:500) and a rabbit anti-PIAS1 pAb (1:500) for 1 h at room temperature. The cells were washed three times with PBS, and incubated with Alexa Fluor 633 goat anti-mouse IgG (H+L) (1:500) and Alexa Fluor 488 goat anti-rabbit IgG (H+L) (1:500) for 1 h. After three washes with PBS, the cells were incubated with DAPI (4’,6-diamidino-2-phenylindole, Thermo Fisher Scientific) for 15 min to stain the nuclei. Images were acquired by using a Leica SP2 confocal system (Leica Microsystems, Wetzlar, Germany).

In a separate experiment to determine the effect of *PIAS1* knockout on the expression of viral NP protein, *PIAS1*_KO A549 cells were infected with WSN (H1N1) virus. At 2, 4, 6, and 8 h p.i., the infected cells were subjected to an immunofluorescence assay and confocal microscopy by following a similar procedure to that described above and by using a mouse anti-NP mAb as the primary antibody and Alexa Fluor 633 goat anti-mouse IgG (H+L) as the secondary antibody.

### SUMOylation assay

To determine whether PIAS1 could be a contributor to the overall increase in SUMOylation during IAV infection, A549 cells grown in 12-well plates were transfected with specific siRNA targeting *PIAS1* or scrambled siRNA for 36 h, and then infected with WSN (H1N1) virus at an MOI of 0.01. The overall cellular SUMOylation level was assessed at 0, 12, and 24 h p.i. by using an anti-SUMO1 or anti-SUMO2/3 pAb. The PIAS1 knockdown efficiency was confirmed by western blotting with an anti-PIAS1 pAb.

To analyze the effect of PIAS1 on the SUMOylation of IAV PB2, PB1, and NP, HEK293T cells grown in 6-well plates were transfected with plasmids for the expression of HA-tagged WSNPB2, WSNPB1, or WSNNP, together with or without Flag-SUMO1, Flag-SUMO2, or Flag-SUMO3, Ubc9-V5, and Myc-PIAS1 or its mutants by using Lipofectamine LTX and Plus Reagents. Whole cell lysates were immunoprecipitated with a mouse anti-HA mAb, or rabbit anti-HA pAb, and analyzed by western blotting with a rabbit anti-PB2 pAb, a mouse anti-PB1 mAb or a rabbit anti-NP pAb, a mouse anti-Flag mAb, a rabbit anti-V5 pAb, a rabbit anti-Myc pAb, and appropriate secondary antibodies.

To determine whether PIASxα mediates the SUMOylation of IAV NP, HEK293T cells grown in 6-well plates were transfected with plasmids for the expression of HA-tagged WSNNP, together with or without Flag-SUMO1, Ubc9-V5, and Myc-tagged PIASxα by using Lipofectamine LTX and Plus Reagents. Whole cell lysates were immunoprecipitated with a rabbit anti-HA pAb, and analyzed by western blotting with a rabbit anti-NP pAb, a mouse anti-Flag mAb, a rabbit anti-V5 pAb, a rabbit anti-Myc pAb, and appropriate secondary antibodies.

### Stability of IAV PB2, PB1, and NP in the presence of exogenous SUMOylation components

To examine whether the stability of IAV PB2, PB1, or NP is affected by exogenously expressed SUMOylation components, HEK293T cells grown in 12-well plates were transfected with plasmids for the expression of WSNPB2, WSNPB1, or WSNNP, Ubc9-V5, Myc-PIAS1, and Flag-SUMO1, Flag-SUMO2, or Flag-SUMO3 by using Lipofectamine LTX and Plus Reagents. To determine whether PIAS1-mediated PB2 degradation occurs through the ubiquitin-proteasome pathway, HEK293T cells grown in 12-well plates were transfected with a combination of plasmids expressing WSNPB2, Flag-SUMO1/2/3, Ubc9-V5, and Myc-PIAS1, a combination of plasmids expressing WSNPB2, Flag-SUMO1/2/3, and Myc-PIAS1, or a combination of plasmids expressing WSNPB2 and Myc-PIAS1 or Myc-PIAS1 mutants, by using Lipofectamine LTX and Plus Reagents. At 36 h post-transfection, the cells were treated with cycloheximide (CHX, MedChemExpress, Shanghai, China) or with CHX and MG132 (Sigma-Aldrich) for 0, 4, and 8 h, lysed with SDS-PAGE loading buffer, and then subjected to western blotting with a mouse anti-PB2, anti-PB1, or anti-NP mAb, a rabbit anti-V5 pAb, a rabbit anti-Myc pAb, and appropriate secondary antibodies.

### Generation of a *Pias1* knockout mouse model

A *Pias1* knockout mouse model was generated by Nanjing Biomedical Research Institute of Nanjing University (Nanjing, China). Most likely due to perinatal death, homozygous *Pias1*^*-/-*^ mice were not obtained. Instead, heterozygous *Pias1*^*+/-*^ mice were successfully generated. Genotyping of wild-type and knockout mice was performed with the following primers: forward primer 5’-TTGTGTGGCTTCACCTAGTA-3’ and reverse primer 5’-CAGAACTTCTAGCACTCATT-3’ for *Pias1*^*+/-*^ mice; forward primer 5’-CAGCTCACTTATGATGGCCA-3’ and reverse primer 5’-GCAGCTGAAAGCTAACCACA-3’ for wild-type mice. All mice were on the C57BL/6J background and were maintained under specific-pathogen-free conditions. All mice used were female and 6 weeks of age.

### Viral replication in *Pias1*^*+/-*^ and wild-type mice

To determine the effect of PIAS1 on the pathogenicity and replication of IAV in vivo, groups of twenty-two *Pias1*^*+/-*^ and wild-type C57BL/6J mice were inoculated intranasally with 1.2×10^4^ PFU of WSN (H1N1) virus. Five mice were euthanized on days 2 and 6 p.i., respectively, and mouse lungs were collected, homogenized, and titrated for infectious virus by means of plaque assays on MDCK cells. The remaining mice were monitored daily for 14 days for body weight loss and mortality, and were humanely euthanized with CO_2_ at the end of the 14-day observation period or when the mouse body weight dropped to 70% of the initial weight during the observation period.

### Plaque assays

Plaque assays were performed as described previously [85]. Briefly, MDCK cells were grown in 12-well plates to 90% confluence, and then infected with 10-fold serial dilutions of virus samples in 1 × MEM for 1.5 h at 37°C. The inoculum was then removed, and the cells were washed with 1 × MEM and overlaid with 1% SeaPlaque agarose (Lonza, Rockland, ME, USA) in 1 × MEM containing 0.3% BSA and 0.4 μg/mL TPCK trypsin (Sigma-Aldrich). After a 48-h incubation, the cells were fixed with 4% PFA and stained with 2.5% crystal violet solution. The number of plaques was counted.

### Statistical analysis

Statistical significance was determined by using the Student’s two-tailed unpaired t test with GraphPad Prism software (GraphPad, San Diego, CA, USA); *P* values of <0.05 were considered significant.

The numerical data used in all figures are included in S1 Data.

## Supporting information

S1 Data Excel spreadsheet containing, in separate sheets, the underlying numerical data and statistical analysis for Figure panels 2A, 2C, 2D, 2F, 2G, 2H, 2I, 2J, 2L, 2M, 4B, 4D, 4E, 4F, 4G, 4H, 4I, 4J, 4K, 4L, 4M, 9C, 9D, and 9E. (XLS)

S1 Fig Interaction of PIAS1 and IAV NP in yeast. Yeast strain Y2HGold was co-transformed with the bait plasmid pGBKT7-AH05NP, containing AH05NP fused to the GAL4-binding domain (BD) (BD-AH05NP), and the prey plasmid pGADT7-PIAS1, which encodes PIAS1 fused to the Gal4-activation domain (AD) (AD-PIAS1). In the case of positive protein-protein interactions, blue colonies will form in the QDO/X/A plates in the presence of X-a-Gal. Co-transformation of pGBKT7-53 encoding the Gal4-BD fused with murine p53 (BD-p53) and pGADT7-T encoding the Gal4-AD fused with SV40 large T-antigen (AD-T) served as a positive control. Co-transformation of pGBKT7-Lam encoding the Gal4-BD fused with lamin (BD-Lam) and AD-T served as a negative control. DDO, SD/−Leu/−Trp; QDO, SD/–Ade/–His/–Leu/–Trp; QDO/X/A, SD/–Ade/–His/–Leu/–Trp/X-a-Gal/AbA.
(TIF)

S2 Fig Interaction of IAV PB2, PB1, PA, and PIAS1 in virus-infected cells. HEK293T cells were transfected for 24 h to express Myc-PIAS1, and were then infected with WSN (H1N1) virus (MOI = 5). At 30 h p.i., cell lysates were immunoprecipitated with a mouse anti-PB2 mAb (A), a mouse anti-PB1 mAb (B) or a mouse anti-PA mAb (C), followed by western blotting with a rabbit anti-Myc pAb (A-C) and a mouse anti-PB2 mAb (A), a mouse anti-PB1 mAb (B) or a mouse anti-PA mAb (C).
(TIF)

S3 Fig Co-IP assay to examine the interaction between PIAS1 and IAV HA, NA, M1, or M2. HEK293T cells were transfected individually or in combination with plasmids expressing Myc-PIAS1 and HA, NA, M1 or M2 of WSN (H1N1) virus. Cell lysates were immunoprecipitated with a mouse anti-Myc mAb, and were then subjected to western blotting with a rabbit anti-Myc pAb (A-D) and a rabbit anti-HA pAb (A), a rabbit anti-NA pAb (B), a rabbit anti-M1 pAb (C) or a rabbit anti-M2 pAb (D), for the detection of PIAS1 and HA, NA, M1 or M2, respectively.
(TIF)

S4 Fig Immunofluorescence assay to visualize viral NP expression in IAV-infected *PIAS1*_KO A549 cells. *PIAS1*_KO or control A549 cells were infected with WSN (H1N1) (MOI = 5) virus. At 2, 4, 6, and 8 h p.i., the infected cells were fixed and stained with a mouse anti-NP mAb, followed by incubation with Alexa Fluor 633 goat anti-mouse IgG (H+L). The nuclei were stained with DAPI.
(TIF)

S5 Fig PIAS1 expression is induced in A549 cells and mice during IAV infection. (A) PIAS1 expression in WSN (H1N1) virus-infected A549 cells. A549 cells were infected with WSN (H1N1) virus (MOI = 0.1). Whole cell lysates were collected at the indicated timepoints and subjected to western blotting with a mouse anti-NP mAb, a rabbit anti-PIAS1 pAb, or a rabbit anti-GAPDH pAb. (B) PIAS1 expression in cells treated with IFN-β. A549 cells were treated with or without 25 pg/mL IFN-β for 24 h, and were then subjected to western blotting with a rabbit anti-PIAS1, anti-MX1, or anti-IFITM3 pAb. The band intensities of PIAS1 (A-B), quantified by using ImageJ software, were normalized to GAPDH and are expressed as relative ratios compared with those of cells at 0 h. (C) Pias1 expression in mice infected with different IAVs. Six-week-old female C57BL/6J mice were inoculated intranasally with 10^5^ PFU of WSN (H1N1), 10^2^ PFU of AH05 (H5N1), 10^5^ PFU of AH13 (H7N9), or 10^6^ PFU of SH13 (H9N2) virus. Mice were euthanized on day 3 post-inoculation, and supernatants of lung homogenates were subjected to western blotting with a rabbit anti-PIAS1 pAb. The band intensities of Pias1 were quantified by using ImageJ software and are expressed as the relative ratio to that of the Gapdh band at the bottom of each panel.
(TIF)

S6 Fig PIAS1 mediates minimal SUMOylation of IAV NP by SUMO1 in comparison to PIASxα. (A) PIASxα interacts with IAV NP in a co-IP assay. HEK293T cells were individually transfected or co-transfected with plasmids expressing V5-WSNNP and Flag-PIASxα. Cell lysates were immunoprecipitated with a mouse anti-V5 mAb, and subjected to western blotting with a rabbit anti-V5 pAb and a rabbit anti-Flag pAb for the detection of WSNNP and PIASxα, respectively. (B-C) PIASxα catalyzes the SUMOylation of IAV NP. HEK293T cells were transfected with plasmids expressing HA-tagged WSNNP, along with or without Flag-SUMO1, Ubc9-V5, and Myc-PIASxα. HEK293T cells transfected to express only Flag-SUMO1, Ubc9-V5, and Myc-PIASxα (B), or only Flag-SUMO1 and Myc-PIASxα (C), served as negative controls. At 36 h post-transfection, cell lysates were immunoprecipitated with a rabbit anti-HA pAb, and were then subjected to western blotting with a rabbit anti-NP pAb and a mouse anti-Flag mAb for the detection of NP and SUMO1, respectively. (D) PIAS1 catalyzes minimal SUMOylation of IAV NP. HEK293T cells were transfected with plasmids expressing HA-tagged WSNNP, along with or without Flag-SUMO1, Ubc9-V5, Myc-PIAS1, and Myc-PIAS1 mutants. HEK293T cells transfected to express only Flag-SUMO1 and Myc-PIAS1 served as a negative control. At 36 h post-transfection, cell lysates were immunoprecipitated with a rabbit anti-HA pAb, and were then subjected to western blotting with a rabbit anti-NP pAb and a mouse anti-Flag mAb for the detection of NP and SUMO1, respectively. Red triangle indicates the corresponding SUMOylated viral protein.
(TIF)
