# Supplementary figures and images for: PIAS1-mediated SUMOylation of influenza A virus PB2 restricts viral replication and virulence
Source: PLoS Pathog. 2022 Apr 4;18(4):e1010446. doi: 10.1371/journal.ppat.1010446 (PMC9009768; doi:10.1371/journal.ppat.1010446)

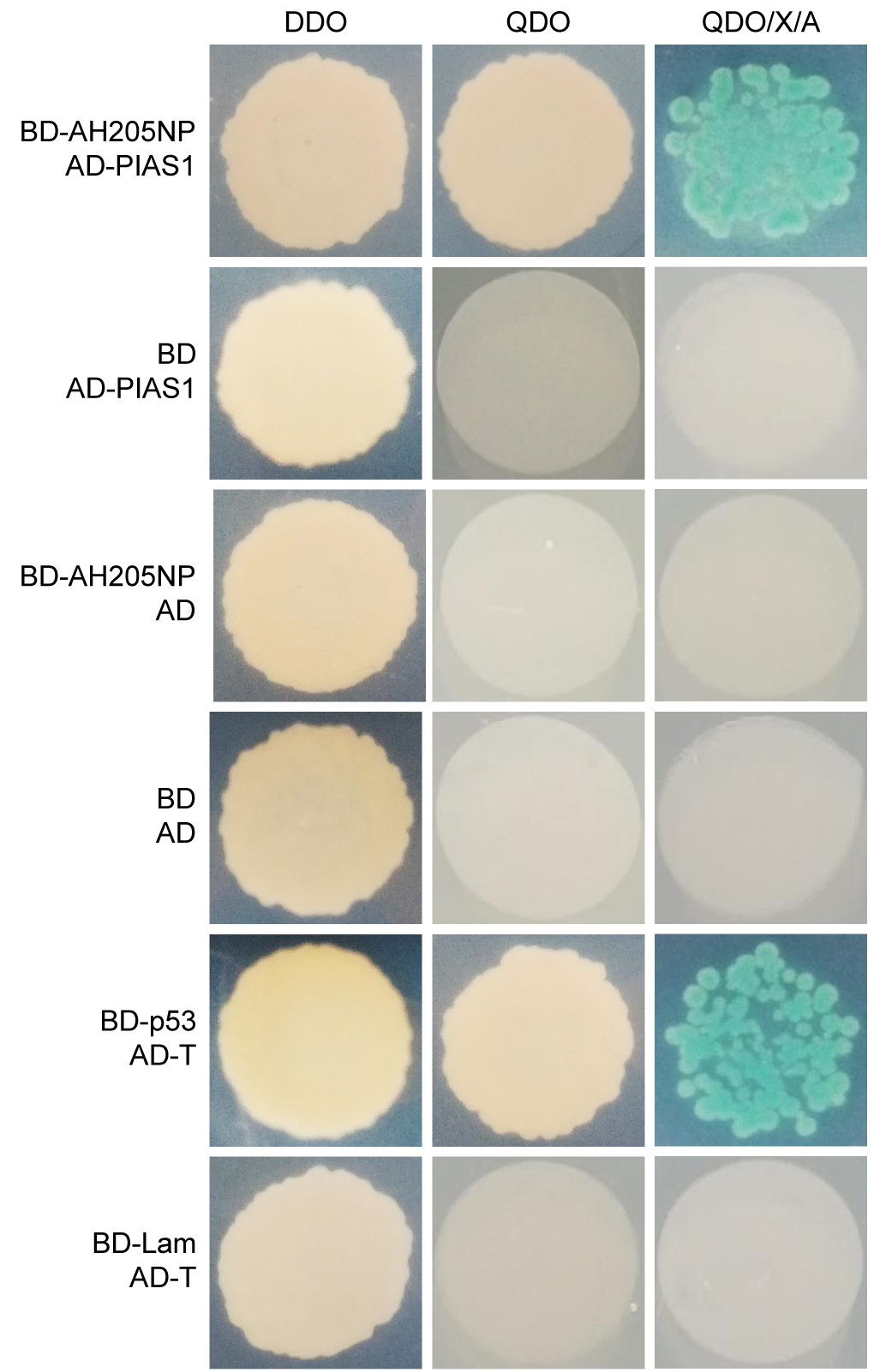

Supplement: S1 Fig — Yeast strain Y2HGold was co-transformed with the bait plasmid pGBKT7-AH05NP, containing AH05NP fused to the GAL4-binding domain (BD) (BD-AH05NP), and the prey plasmid pGADT7-PIAS1, which encodes PIAS1 fused to the Gal4-activation domain (AD) (AD-PIAS1). In the case of positive protein-protein interactions, blue colonies will form in the QDO/X/A plates in the presence of X-a-Gal. Co-transformation of pGBKT7-53 encoding the Gal4-BD fused with murine p53 (BD-p53) and pGADT7-T encoding the Gal4-AD fused with SV40 large T-antigen (AD-T) served as a positive control. Co-transformation of pGBKT7-Lam encoding the Gal4-BD fused with lamin (BD-Lam) and AD-T served as a negative control. DDO, SD/−Leu/−Trp; QDO, SD/–Ade/–His/–Leu/–Trp; QDO/X/A, SD/–Ade/–His/–Leu/–Trp/X-a-Gal/AbA. (TIF) [file ppat.1010446.s002.tif]

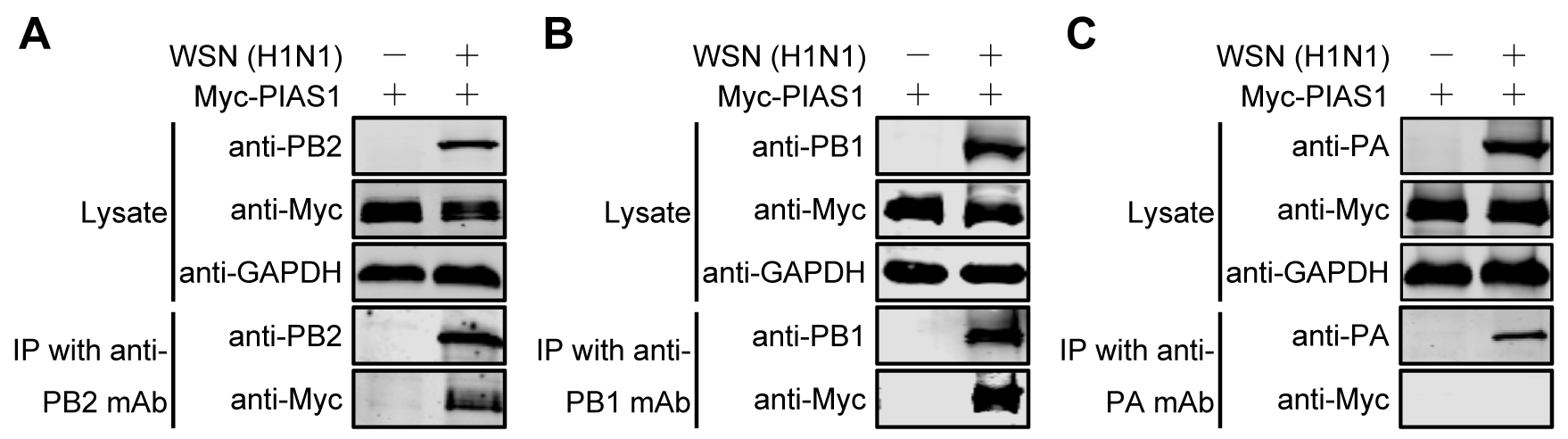

Supplement: S2 Fig — HEK293T cells were transfected for 24 h to express Myc-PIAS1, and were then infected with WSN (H1N1) virus (MOI = 5). At 30 h p.i., cell lysates were immunoprecipitated with a mouse anti-PB2 mAb (A), a mouse anti-PB1 mAb (B) or a mouse anti-PA mAb (C), followed by western blotting with a rabbit anti-Myc pAb (A-C) and a mouse anti-PB2 mAb (A), a mouse anti-PB1 mAb (B) or a mouse anti-PA mAb (C). (TIF) [file ppat.1010446.s003.tif]

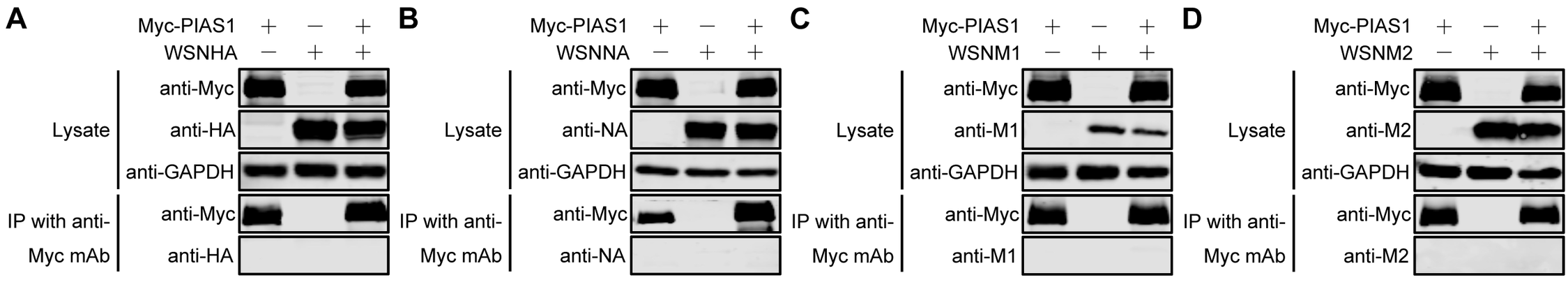

Supplement: S3 Fig — HEK293T cells were transfected individually or in combination with plasmids expressing Myc-PIAS1 and HA, NA, M1 or M2 of WSN (H1N1) virus. Cell lysates were immunoprecipitated with a mouse anti-Myc mAb, and were then subjected to western blotting with a rabbit anti-Myc pAb (A-D) and a rabbit anti-HA pAb (A), a rabbit anti-NA pAb (B), a rabbit anti-M1 pAb (C) or a rabbit anti-M2 pAb (D), for the detection of PIAS1 and HA, NA, M1 or M2, respectively. (TIF) [file ppat.1010446.s004.tif]

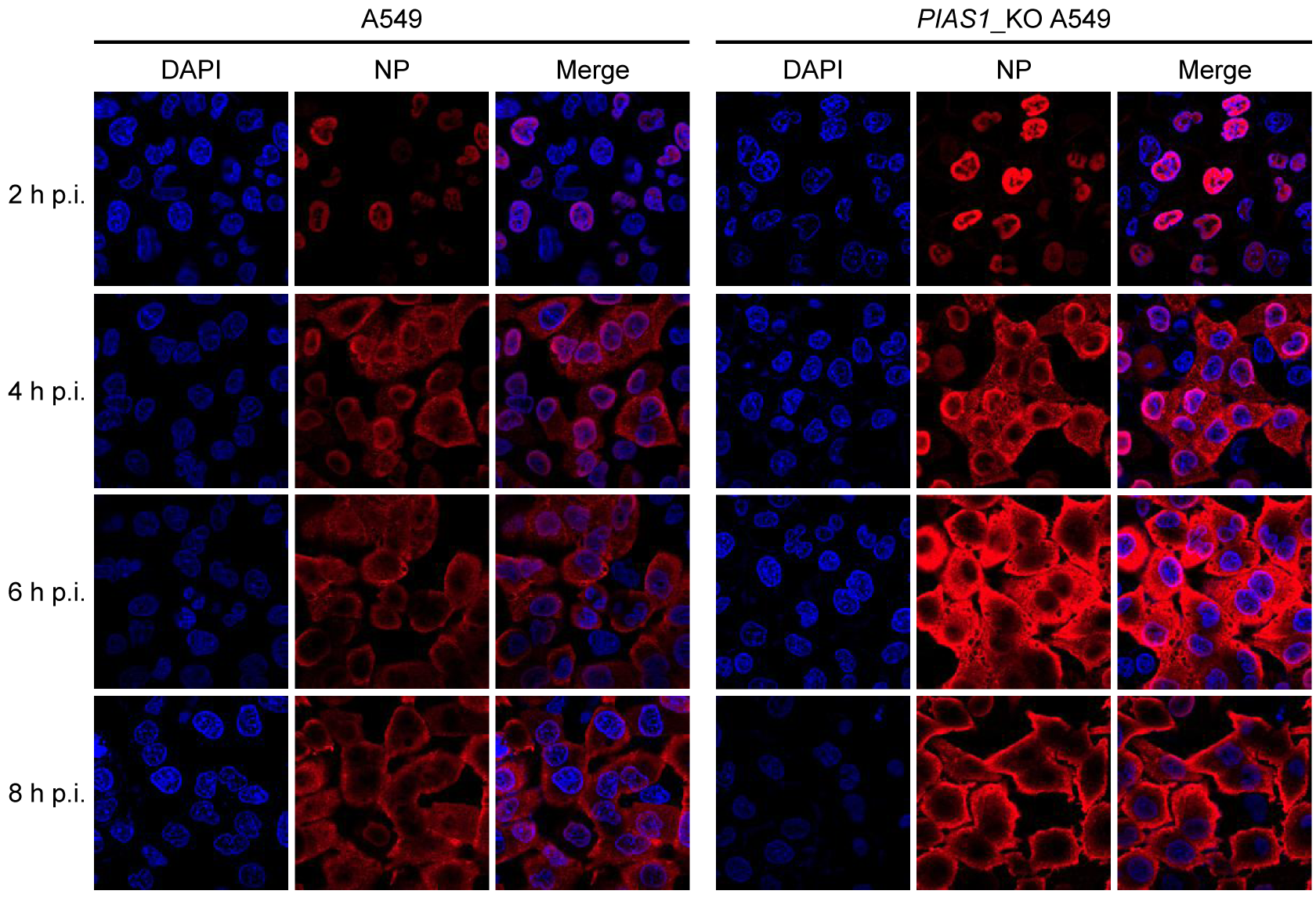

Supplement: S4 Fig — PIAS1_KO or control A549 cells were infected with WSN (H1N1) (MOI = 5) virus. At 2, 4, 6, and 8 h p.i., the infected cells were fixed and stained with a mouse anti-NP mAb, followed by incubation with Alexa Fluor 633 goat anti-mouse IgG (H+L). The nuclei were stained with DAPI. (TIF) [file ppat.1010446.s005.tif]

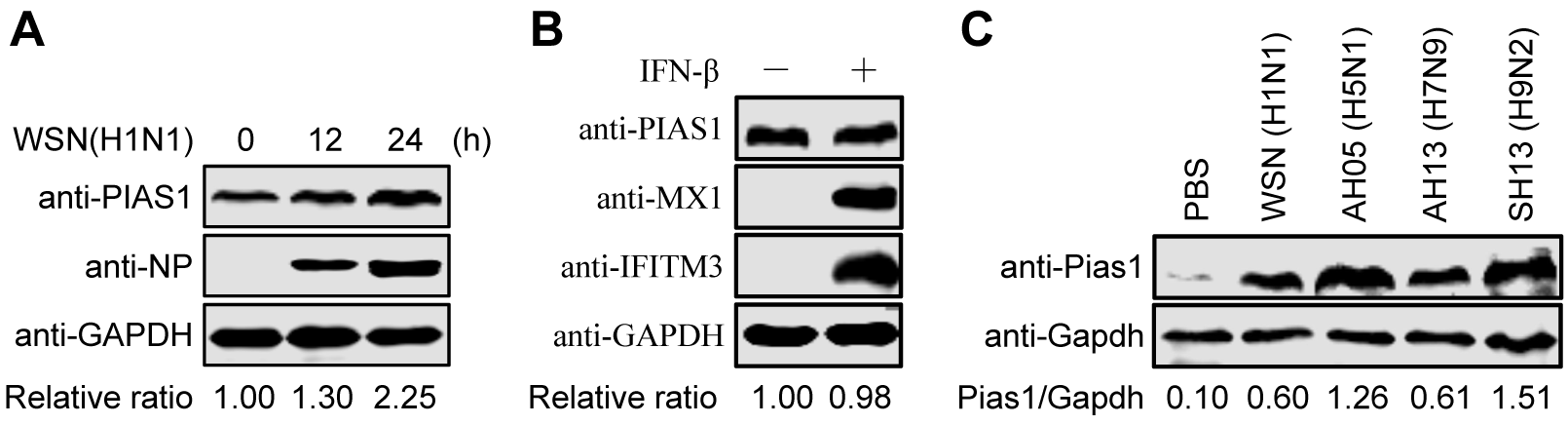

Supplement: S5 Fig — (A) PIAS1 expression in WSN (H1N1) virus-infected A549 cells. A549 cells were infected with WSN (H1N1) virus (MOI = 0.1). Whole cell lysates were collected at the indicated timepoints and subjected to western blotting with a mouse anti-NP mAb, a rabbit anti-PIAS1 pAb, or a rabbit anti-GAPDH pAb. (B) PIAS1 expression in cells treated with IFN-β. A549 cells were treated with or without 25 pg/mL IFN-β for 24 h, and were then subjected to western blotting with a rabbit anti-PIAS1, anti-MX1, or anti-IFITM3 pAb. The band intensities of PIAS1 (A-B), quantified by using ImageJ software, were normalized to GAPDH and are expressed as relative ratios compared with those of cells at 0 h. (C) Pias1 expression in mice infected with different IAVs. Six-week-old female C57BL/6J mice were inoculated intranasally with 105 PFU of WSN (H1N1), 102 PFU of AH05 (H5N1), 105 PFU of AH13 (H7N9), or 106 PFU of SH13 (H9N2) virus. Mice were euthanized on day 3 post-inoculation, and supernatants of lung homogenates were subjected to western blotting with a rabbit anti-PIAS1 pAb. The band intensities of Pias1 were quantified by using ImageJ software and are expressed as the relative ratio to that of the Gapdh band at the bottom of each panel. (TIF) [file ppat.1010446.s006.tif]

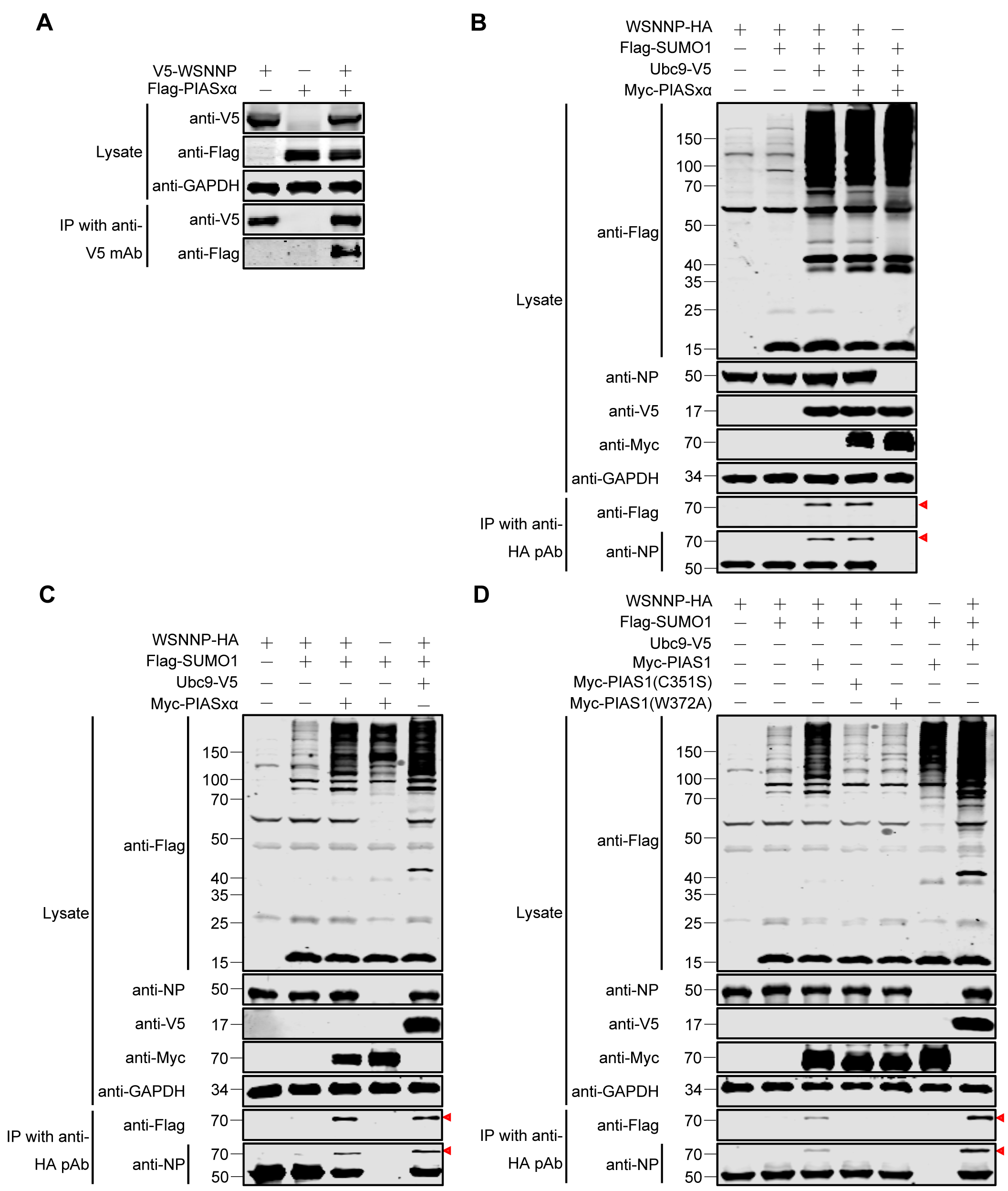

Supplement: S6 Fig — (A) PIASxα interacts with IAV NP in a co-IP assay. HEK293T cells were individually transfected or co-transfected with plasmids expressing V5-WSNNP and Flag-PIASxα. Cell lysates were immunoprecipitated with a mouse anti-V5 mAb, and subjected to western blotting with a rabbit anti-V5 pAb and a rabbit anti-Flag pAb for the detection of WSNNP and PIASxα, respectively. (B-C) PIASxα catalyzes the SUMOylation of IAV NP. HEK293T cells were transfected with plasmids expressing HA-tagged WSNNP, along with or without Flag-SUMO1, Ubc9-V5, and Myc-PIASxα. HEK293T cells transfected to express only Flag-SUMO1, Ubc9-V5, and Myc-PIASxα (B), or only Flag-SUMO1 and Myc-PIASxα (C), served as negative controls. At 36 h post-transfection, cell lysates were immunoprecipitated with a rabbit anti-HA pAb, and were then subjected to western blotting with a rabbit anti-NP pAb and a mouse anti-Flag mAb for the detection of NP and SUMO1, respectively. (D) PIAS1 catalyzes minimal SUMOylation of IAV NP. HEK293T cells were transfected with plasmids expressing HA-tagged WSNNP, along with or without Flag-SUMO1, Ubc9-V5, Myc-PIAS1, and Myc-PIAS1 mutants. HEK293T cells transfected to express only Flag-SUMO1 and Myc-PIAS1 served as a negative control. At 36 h post-transfection, cell lysates were immunoprecipitated with a rabbit anti-HA pAb, and were then subjected to western blotting with a rabbit anti-NP pAb and a mouse anti-Flag mAb for the detection of NP and SUMO1, respectively. Red triangle indicates the corresponding SUMOylated viral protein. (TIF) [file ppat.1010446.s007.tif]
